# Anatomical insights into fish terrestrial locomotion: A study of barred mudskipper (*Periophthalmus argentilineatus*) fins based on μCT 3D reconstructions

**DOI:** 10.1111/joa.14071

**Published:** 2024-06-06

**Authors:** Fabienne Ziadi‐Künzli, Ken Maeda, Pavel Puchenkov, Mahesh M. Bandi

**Affiliations:** ^1^ Nonlinear and Non‐equilibrium Physics Unit Okinawa Institute of Science and Technology Graduate University Okinawa Japan; ^2^ Marine Eco‐Evo‐Devo Unit Okinawa Institute of Science and Technology Graduate University Okinawa Japan; ^3^ Scientific Computing & Data Analysis Section Okinawa Institute of Science and Technology Graduate University Okinawa Japan

**Keywords:** gobies, microcomputed tomography, mudskipper locomotion, pectoral fin, ray‐finned fish, terrestriality

## Abstract

Mudskippers are a group of extant ray‐finned fishes with an amphibious lifestyle and serve as exemplars for understanding the evolution of amphibious capabilities in teleosts. A comprehensive anatomical profile of both the soft and hard tissues within their propulsive fins is essential for advancing our understanding of terrestrial locomotor adaptations in fish. Despite the ecological significance of mudskippers, detailed data on their musculoskeletal anatomy remains limited. In the present research, we utilized contrast‐enhanced high‐resolution microcomputed tomography (μCT) imaging to investigate the barred mudskipper, *Periophthalmus argentilineatus*. This technique enabled detailed reconstruction and quantification of the morphological details of the pectoral, pelvic, and caudal fins of this terrestrial mudskipper, facilitating comparison with its aquatic relatives. Our findings reveal that *P. argentilineatus* has undergone complex musculoskeletal adaptations for terrestrial movement, including an increase in muscle complexity and muscle volume, as well as the development of specialized structures like aponeuroses for pectoral fin extension. Skeletal modifications are also evident, with features such as a reinforced shoulder‐pelvic joint and thickened fin rays. These evolutionary modifications suggest biomechanically advanced fins capable of overcoming the gravitational challenges of terrestrial habitats, indicating a strong selective advantage for these features in land‐based environments. The unique musculoskeletal modifications in the fins of mudskippers like *P. argentilineatus*, compared with their aquatic counterparts, mark a critical evolutionary shift toward terrestrial adaptations. This study not only sheds light on the specific anatomical changes facilitating this transition but also offers broader insights into the early evolutionary mechanisms of terrestrial locomotion, potentially mirroring the transformative journey from aquatic to terrestrial life in the lineage leading to tetrapods.

## Graphical abstract

This study reveals that the barred mudskipper, *Periophthalmus argentilineatus*, has evolved intricate musculoskeletal features in its fins, such as increased muscle volume, skeletal reinforcements, and specialized aponeuroses, signifying a profound evolutionary shift toward terrestrial life.

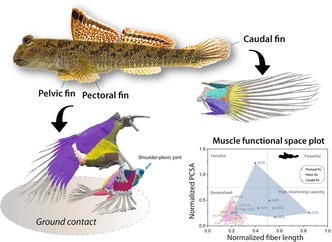

## 1 INTRODUCTION

**FIGURE 1:**
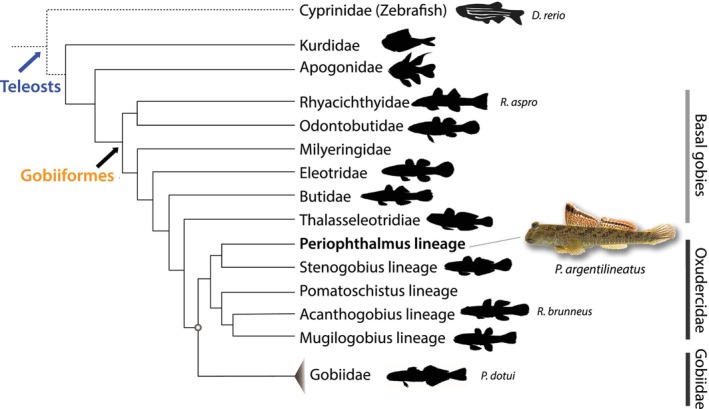
Backbone tree of gobies (Gobiiformes) representing a diversified fish lineage with over 2000 species. Two major groups are recognized as derived goby: Oxudercidae and Gobiidae (Agorreta et al., 2013; Jeon et al., 2021; McCraney et al., 2020). Mudskippers represent the Periophthalmus lineage within Oxudercidae and comprise 10 genera with over 43 species, including eel gobies. Zebrafish silhouette image courtesy of phylopic.org.

Mudskippers (Class Osteichthyes, Order Gobiiformes, Family Oxudercidae; Figure 1) are semi‐terrestrial, air‐breathing actinopterygian fish that offer a unique perspective into evolutionary adaptations linked to the transition from aquatic to terrestrial environments (Jaafar & Murdy, 2017; Nelson et al., 2016). Originating during the late Eocene epoch, around 40 to 29 million years ago (Mya) (Thacker, 2015), these species inhabit swamps, estuaries, and other intertidal ecosystems throughout the tropical Indo‐West Pacific. Mudskippers exhibit an impressive array of specializations, inter alia, in their ecology, morphology, genetics, and physiology, reflecting their dual adaptation to both aquatic and terrestrial environments.

For example, they possess bulging eyes located dorsally that are adapted for more accurate vision in air (Huby et al., 2021; You et al., 2014). Mudskippers can breathe through their skin and the lining of their mouth and throat when these surfaces are moist, facilitating direct atmospheric gas exchange (Beon et al., 2013; Damsgaard et al., 2020; Graham et al., 2007). Maintenance of hydration is critical for mudskippers, not only for their general survival but also to facilitate their unique cutaneous and buccopharyngeal respiration (Hidayat et al., 2022). On land, mudskippers use their protrusible mouths to effectively capture prey, contrasting with their aquatic behavior where they exhibit typical fish suction feeding (Sponder & Lauder, 1981). The most remarkable adaptation of mudskippers, however, is their terrestrial mobility. They utilize their robust, muscular fins for “walking” or “skipping” across mudflats and climbing obstacles, demonstrating locomotor kinematics that are distinct from those of fully aquatic fish (Katayama et al., 2019; Pace & Gibb, 2014; Quigley et al., 2022).

Although terrestrial locomotion behaviors have appeared independently in various fish species across the actinopterygian evolutionary tree (Lutek et al., 2022; Ord & Cooke, 2016; Wright & Turko, 2016), only mudskippers exhibit a sustained mode of terrestrial locomotion, termed “crutching.” This specialized form of locomotion method involves the mudskipper keeping its body axis relatively static on the substrate, while synchronously moving their paired pectoral fins forward in conjunction with their pelvic fins. Crutching is a rather slow‐speed locomotor gait but can be complemented with tail use on inclines (McInroe et al., 2016; Naylor & Kawano, 2022) or by spurts of axial undulation (Animalogic, 2021, 3:03). Overall, mudskipper appendage‐based locomotion mode contrasts with other forms of amphibious movements found in ray‐finned fishes and appears to be exclusive to mudskippers (Lutek et al., 2022; Pace & Gibb, 2009).

Species within the genus *Periophthalmus* have further adapted their crutching locomotion mode technique. This is characterized by a reduction in the contact area through body arching and a distinct angling of the caudal fin away from the substrate at ~ 45°. During the propulsive phase of “arched” terrestrial crutching, these modifications lead to a tripedal stance, with the points of contact being the pectoral fins and the tail, effectively forming three “legs” (McInroe et al., 2016; Pace & Gibb, 2009; van Dijk, 1960).

In addition to crutching during steady “walking” on land, mudskippers employ an escape response, or skipping, when threatened (Harris, 1960; Macnae, 1969). The terrestrial escape response of mudskippers varies from the typical aquatic C‐start escape response of fish in the water, introducing a longer preparatory phase through acute axial skeleton flexion posteriorly so that the tail is positioned near the head (Domenici & Blake, 1997). The propulsive phase involves a final push‐off from the ground, enabled by body straightening (Swanson & Gibb, 2004). This behavior tends to propel the fish in the same direction it was originally facing. The result is a swift movement away from the perceived threats.

Given the crucial role of the tail in providing the necessary thrust for terrestrial escape in mudskippers, an adaptation toward a stronger tail region could have enhanced their terrestrial locomotion performance without compromising vertebral flexibility, essential for their unique preparatory phase of the escape response (Ghanbarifardi et al., 2020; Tran et al., 2023). Therefore, a balance between tail strength and vertebral flexibility may represent a significant morphological adaptation in response to the terrestrial challenges faced by mudskippers.

Although advancements have been made in understanding mudskippers' terrestrial adaptations, there is a lack of a comprehensive 3D anatomical map in the current literature detailing the skeletal and muscular organization of mudskippers' locomotor appendages. These appendages act as integrative functional linkages within the vertebral body, orchestrating coordinated movements that allow mudskippers to navigate in vastly different environments of water and land. The last in‐depth studies examining the anatomical features of goby fins, including those of mudskippers, were conducted in the early to mid‐20th century (Eggert, 1929; Harris, 1960). Given the rapid advances in technology and the growing focus on terrestrial adaptations in aquatic species (Sayer, 2005; Sayer & Davenport, 1991; Standen et al., 2014; Wilhelm et al., 2015), a thorough reassessment is both timely and necessary for our understanding of the structural layout of amphibious behavior in fish.

**FIGURE 2:**
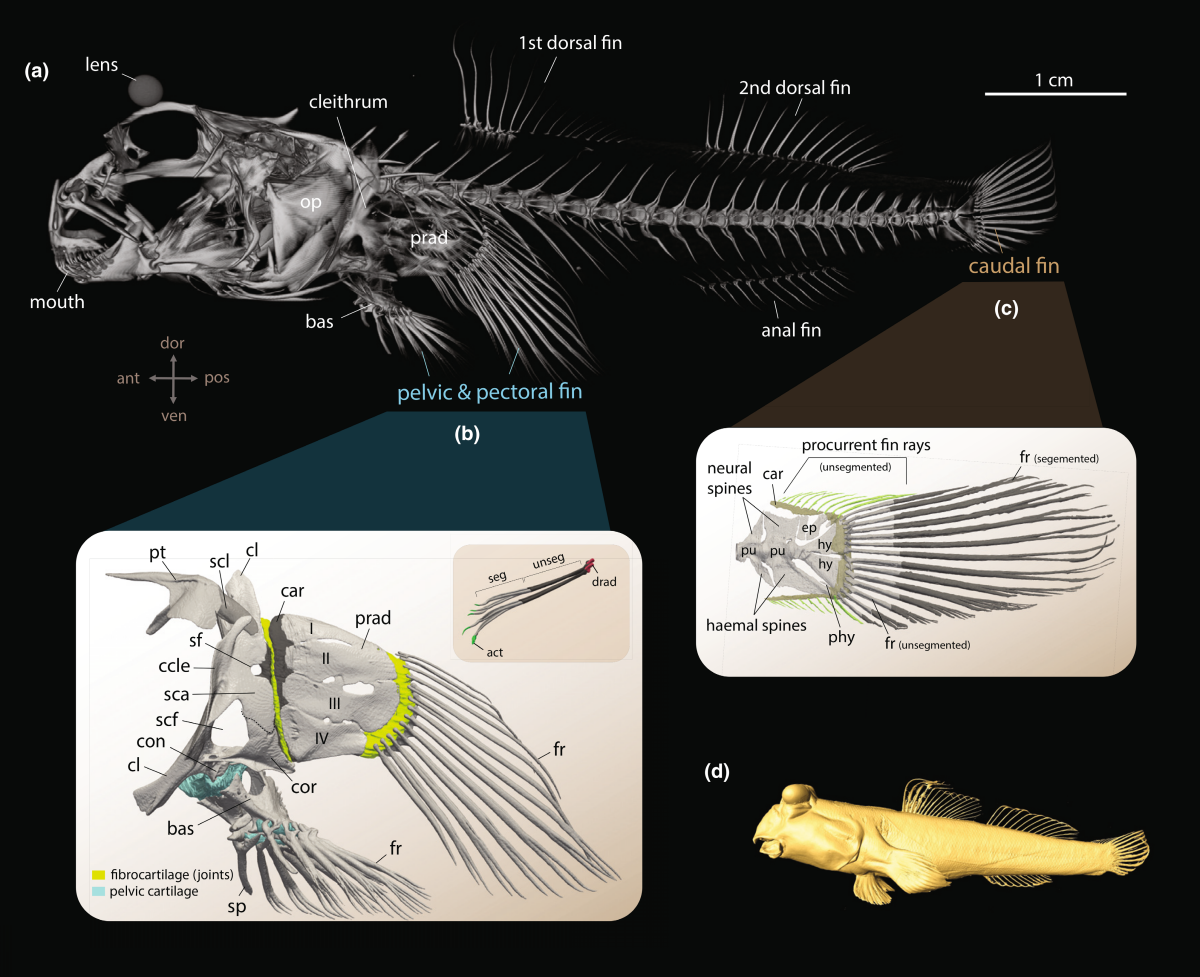
Overview of osteological features of employed fins for terrestrial locomotion in *Periophthalmus argentilineatus*. (a) Volume rendered μCT image showing the mudskipper skeletal system. (b) All bony fishes have a pair of laterally oriented pectoral fins behind the operculum (op) with a pectoral girdle that consists of endochondral skeletal elements supporting the dermal fin rays (fr). Teleosts usually have four proximal radials (prad) that articulate through fibrocartilaginous pads proximally with the cleithrum (cl) and distally with the fin rays (Enny et al., 2020). The pelvic fins in fishes are paired appendages on the ventral part of the body wall with bony pelvic plates (basipterygium, bas), a reduced number of radials at the fin base and the fin rays (fr). Pectoral and pelvic fins are homologous to the tetrapod fore and hindlimb, respectively (Mercader, 2007). Inset in (b) illustrates the features of pectoral fin rays in mudskippers: distal radials (drad) are attached to the fin ray base. Each fin ray is made up of an unsegmented part proximally (unseg, dark gray) and a segmented part distally (seg, gray). The tip of the fin ray contains actinotrichia collagens (act). (c) As representatives of modern teleosts, gobies possess a homocercal (symmetrical) tail where the vertebrae do not extend into a lobe (Lauder, 2000). The distal part of the caudal fin rays (fr) is segmented (dark gray). Procurrent fin rays (green) are unsegmented. (b, c) Manual segmentation of fin bone and cartilage from μCT images, lateral view. (d) 3D visualization of the mudskipper based on isosurface construction (same specimen as in (a)). act, actinotrichia; bas, basipterygium; car, cartilage; cl, cleithrum; ccle, crista cleithralis externa; con, ondyle; cor, coracoid; ep, epural; hy, hypural plate; fr, fin ray; op, operculum; prad, proximal radial; phy, parhypural; pt, post‐temporal; pu, pleural vertebra; sca, scapula; scf, scapulocoracoid fenestra; sf, scapular fenestra; scl, supracleithrum; sp, spine.

In this article, we utilize advanced iodine‐based microcomputed X‐ray tomography (μCT) to create three‐dimensional (3D) reconstructions, allowing for detailed mapping of the musculoskeletal system in the fins of barred mudskipper, *Periophthalmus argentilineatus* (Figure 2). Contrast‐enhancing and high‐resolution images allow us to closely examine the soft tissues, such as tendons, ligaments, and muscles, as well as calcified bones that provide functional linkages among the vertebral body's structural elements during coordinated movements in the water and on land (Altringham & Ellerby, 1999; Gillis & Blob, 2001).

An additional objective of this study is to delineate the roles of specific fin muscles—encompassing all pectoral, pelvic, and intrinsic caudal fin muscles—in terrestrial locomotion. This includes a detailed evaluation of their origins, insertions onto the skeleton, and overall properties to clarify their roles and their ability to generate muscle force on land.

By integrating the anatomical details and features of the appendicular fin system using 3D imaging techniques, we expect our review of mudskipper locomotor structures will aid future research on the evolution of fin muscles in fishes with amphibious behavior. This data set will have intrinsic value in applications to other ray‐finned fishes as well.

## 2 MATERIALS AND METHODS

### 2.1 Sample selection and specimen preparation

Given the considerable changes in fin skeletal and muscular morphology observed within the *Periophthalmus* lineage (Eggert, 1929; Ghanbarifardi et al., 2020; Harris, 1960; Murdy, 1989; Okamoto et al., 2018), our study analyzed μCT scans from four mudskipper specimens along with additional goby samples, including a zebrafish (as a representative of a more ancestral teleost form), to explore our hypotheses concerning the homology of fin muscles among these species (Figure 1; Table 1).

**TABLE 1:** Specimens list and summary of μCT scanning parameters used for this study.

Species	Total length in mm (sex)	Habitat	Main locomotion type	Voxel size (μm)	Optical magnitude	Exposure time (s)	Voltage/power (kV/W)
*Periophthalmus argentilineatus*	116.8 (♀)	Mangrove	Crutching, skipping	24.15 (12.26)	0.39 (0.39)	2 (1.5)	100/9 (80/7)
	88.5 (♂)	Mangrove	Crutching, skipping	26.81	0.39	1	80/7
	82.7 (♀)	Mangrove	Crutching, skipping	18.04	0.39	1	80/7
	66.8 (♂)	Mangrove	Crutching, skipping	22.12 (4.31)	0.39 (4)	2 (2)	100/9 (80/7)
*Rhyacichthys aspro*	59.8 (—)	Freshwater stream	Burst‐and‐coast	23.12 (9.24)	0.39 (0.39)	1 (1.5)	90/8 (80/7)
*Rhinogobius brunneus*	38.8 (♂)	Freshwater stream	Burst‐and‐coast	13.69 (4.03)	0.39 (4)	1 (1)	90/8 (80/7)
*Parioglossus dotui*	24.6 (♀)	Estuary	Free swimming, station‐holding	9.15 (4.65)	0.39 (4)	1 (1.5)	90/8 (80/7)
*Danio rerio*	29.2 (♀)	—	Free swimming, station‐holding	12.82 (8.24)	0.39 (0.39)	1.5 (1.5)	90/8 (90/8)

*Note*: Scanning parameters for close‐up scans of dissected fins are indicated in parentheses.

In this context, the term “homology” with respect to fin muscles in gobies is used in the phylogenetic sense, as defined by Patterson (1988), implying synapomorphy or shared derived characteristics (see also Abdala & Diogo, 2010; Diogo & Abdala, 2010). This approach to homology considers muscle characters through an analysis of common origin, inferred by examining their function, topology, and ontogeny.

*Rhyacichthys aspro* (Rhyacichthyidae) represents a basal goby, while *Rhinogobius brunneus* (Acanthogobius lineage) and *Parioglossus dotui* (Gobiidae) are derived gobies (Figure 1; Table 1). All the material used in this study was collected by field sampling on Okinawa Island, Japan. A specimen of zebrafish was purchased from a local pet shop.

We confirmed the sex of all fish by inspecting μCT images of their reproductive organs. Additionally, for mudskippers, we differentiated sexes by examining the shape of the genital papilla, which is triangular in males and heart‐shaped in females. All fish were mature except for *R. aspro*, which was immature.

Fish specimens were euthanized with 2‐phenoxyethanol solution and subsequently fixed in 10% formalin and later stored in 70%–99% ethanol. The procedures used to handle fish specimens in this study were approved by the Animal Care and Use Committees of the Okinawa Institute of Science and Technology Graduate University (2017‐196 and 2020‐287).

The musculoskeletal terminology follows Winterbottom (1973). The individual fin muscle action and potential range of movement were compared with available literature for ray‐finned fishes and inferred by observing muscle origin, its insertion, and fiber direction.

### 2.2 Microcomputed tomography

Muscle tissue and cartilaginous material can successfully be imaged using iodine as a staining agent (Jeffery et al., 2011; Kupczik et al., 2015; Metscher, 2009). Prior to scanning, each sample was placed in an iodine‐based contrast stain solution and stored at room temperature. The staining agent contained 2 g elemental iodine dissolved in 50 mL absolute ethanol (I_2_‐ethanol).

For whole‐body scans, the material was allowed to uptake the staining solution for at least 2 days. To obtain a high resolution of the pectoral fin musculature, we performed close‐up scans of the dissected left pectoral fin of selected specimens (Table 1). Dissected parts were stained for a minimum of 24 h. Prior to scanning, each sample was washed repeatedly with 99% ethanol and wrapped in cling film or wet paper to prevent dehydration during scanning. The sample was placed in a plastic container slightly larger than the specimen and fixed with paper material to prevent movement artifacts during the scan.

For imaging of the internal morphology of the fins, we employed a commercially available microcomputed tomography system (Zeiss Xradia Versa 510) at OIST with an X‐ray source operating at an anode voltage at 80–100 kV and power ranging between 7 and 9 W. We used standard objectives (0.39×, 4×) and different combinations of source sample and sample‐detector distances to fit and optimize the entire region of interest. Images were acquired with an exposure time of 1–2 s per projection, intensities <30,000, and a total of 1601 projections over 360°.

The vertical stitching feature was applied to join multiple tomographies. Serial scans were performed on the same fish samples with isotropic nominal voxel sizes ranging between 4.0–24.1 μm (Table 1). The projection data from each scan were reconstructed using the integrated volume reconstruction software of the Xradia machine.

The resulting reconstructions were exported in .txm or DICOM format and imported into AMIRA (version 6.5, Thermo Fisher Scientific, Waltham, Massachusetts, USA) for manual segmentation of anatomical structures (bone, cartilaginous tissue, muscles, fascia, tendons, etc.) and to render isosurfaces from the volumetric data. Using global thresholding on a gray‐scale range of 14,000–30,000, manual annotation of structures was performed with the brush tool or magic wand tool on every fourth slice and then interpolated. The surface generation algorithm implemented in the *Generate Surface* module was applied for each material using smoothing function values <2.5. The resulting 3D surface meshes were exported in .ply format and imported into the ParaView package for data visualization (Ayachit, 2015). All figures were created with Adobe Illustrator (Adobe Inc., 2019, available at: https://adobe.com/products/illustrator).

### 2.3 Fin shape

The shapes of pectoral, pelvic, and caudal fins were determined by the relative lengths of their respective fin rays. We manually segmented fin rays in samples of *P. argentilineatus* (*n* = 4) to represent mudskippers, and in *R. brunneus* (*n* = 1), as a representative of aquatic gobies (Table 1). Surfaces were exported as .ply files into Checkpoint software (version 2020.10.13, Stratovan Checkpoint, Davis, CA, USA) to measure the length of each fin ray by applying curve points. The means of fin ray lengths were plotted to visualize fin shape for samples of both species. Fin ray length measurements were standardized beforehand relative to the total length of the respective specimen (Table 1).

### 2.4 Video animation

For a comprehensive understanding and to better visualize the skeletal structure of each fin alongside its associated locomotor muscle, we have produced an animated video representation (Video S1). Each segmented model of the bones and muscles was exported from the segmentation software in common *.stl file format. The models then were imported into 3D software, – Blender (Community, 2018) (available at http://www.blender.org). The animation has been described using python code and its implementation in Blender's API, by defining the time duration for showing each muscle, groups of muscles, transitions, and titles. The camera rig was made and animated manually, following the muscle appearance in the 3D scene. Programming this animation in python helped to link the legend and render it as a separate layer which was composed over the 3D rendered frames. The labels of the muscles and their snapping to the specific points on the 3D models of muscles and bones were designed using vector math and then programmed using build‐in blender tools and python. Finally, all the frames were rendered, the layers composed, and all that got compacted into the video file using open‐source ffmpeg program (Tomar, 2006).

### 2.5 Physiological cross‐sectional area

Skeletal muscle architecture is defined as the geometric properties of a muscle and the arrangement of its fibers within, the knowledge of which functionally determines the muscle performance capacity (Gans, 1982; Powell et al., 1984). Muscle properties such as the fiber length (*L*_f_) and the physiological cross‐sectional area (PCSA) are especially important parameters of muscle architecture and serve as proxies to infer muscle function. Fiber length reflects the excursion or “working range” of a muscle, whereas the PCSA is directly proportional to the force‐generating capacities of a given muscle.

PCSA can be used to compare muscle force production between species and to explore the trade‐offs between potential force generation (PCSA) and working range capacities (Allen et al., 2010, 2014; Dick & Clemente, 2017; Martin et al., 2020). Here, we use PCSA as an indirect indicator to compare inferred muscle force production among different fins (i.e., pectoral, pelvic, caudal) within the same species.

PCSA of a muscle can be estimated from the muscle volume (*V*_m_) divided by the mean muscle fiber length (PCSA = *V*_m_/*L*_f_). We did not observe pennate muscles, which could influence PCSA because of the angle of muscle fibers relative to the force‐generating axis. Therefore, we did not need to account for the complexities introduced by pennate muscle arrangements.

To determine fiber length, we manually segmented 5–10 fibers for each muscle using the interpolation function in AMIRA. We then exported the surface mesh to Checkpoint software (version 2020.10.13, Stratovan Checkpoint, Davis, CA, USA) to measure the length of each fiber by placing curve points along the structure. The mean fiber length for each muscle was calculated and used for subsequent calculation of the PCSA.

Using geometric similarity for length, area, and volume (Dick & Clemente, 2017), muscle fiber length was normalized to *V*_body_^1/3^, PCSA was normalized to *V*_body_^2/3^, and muscle volume was normalized to *V*_body_ (Allen et al., 2010). Normalization of fish body volume (*V*_body_) allows for comparison between specimens of different sizes.

Mean normalized PCSA values were then plotted against mean normalized fiber lengths to generate a functional performance space for pectoral, pelvic, and caudal fin muscles of *P. argentilineatus*. Since PCSA is proportional to the inferred maximum muscle force and fiber length is indicative of muscle excursion, this functional space plot produces a visualization of the relative forces and excursions of muscles thereby giving an estimation of relative muscle function based on their position in the biplot (Allen et al., 2010).

There is a structure–function trade‐off between PCSA and fiber length for a muscle with a given volume. Increased PCSA (maximizing muscle force) comes at the cost of reduced fiber lengths (smaller muscle excursion and contraction velocity) and vice versa. In addition, “powerful” muscles have a large volume and are metabolically expensive.

## 3 RESULTS

### 3.1 Pectoral fin

#### 3.1.1 Skeletal fin anatomy

The pectoral fin skeleton of *P. argentilineatus* is composed of a well‐built shoulder girdle with a prominent crista cleithralis externa along the anterior border of the cleithrum, and an enlarged scapula and coracoid for locomotor muscle attachment (Figure 2b). Ventrally, a condyle protrudes from the shoulder and articulates with the pelvic girdle (see also pelvic fin).

**FIGURE 3:**
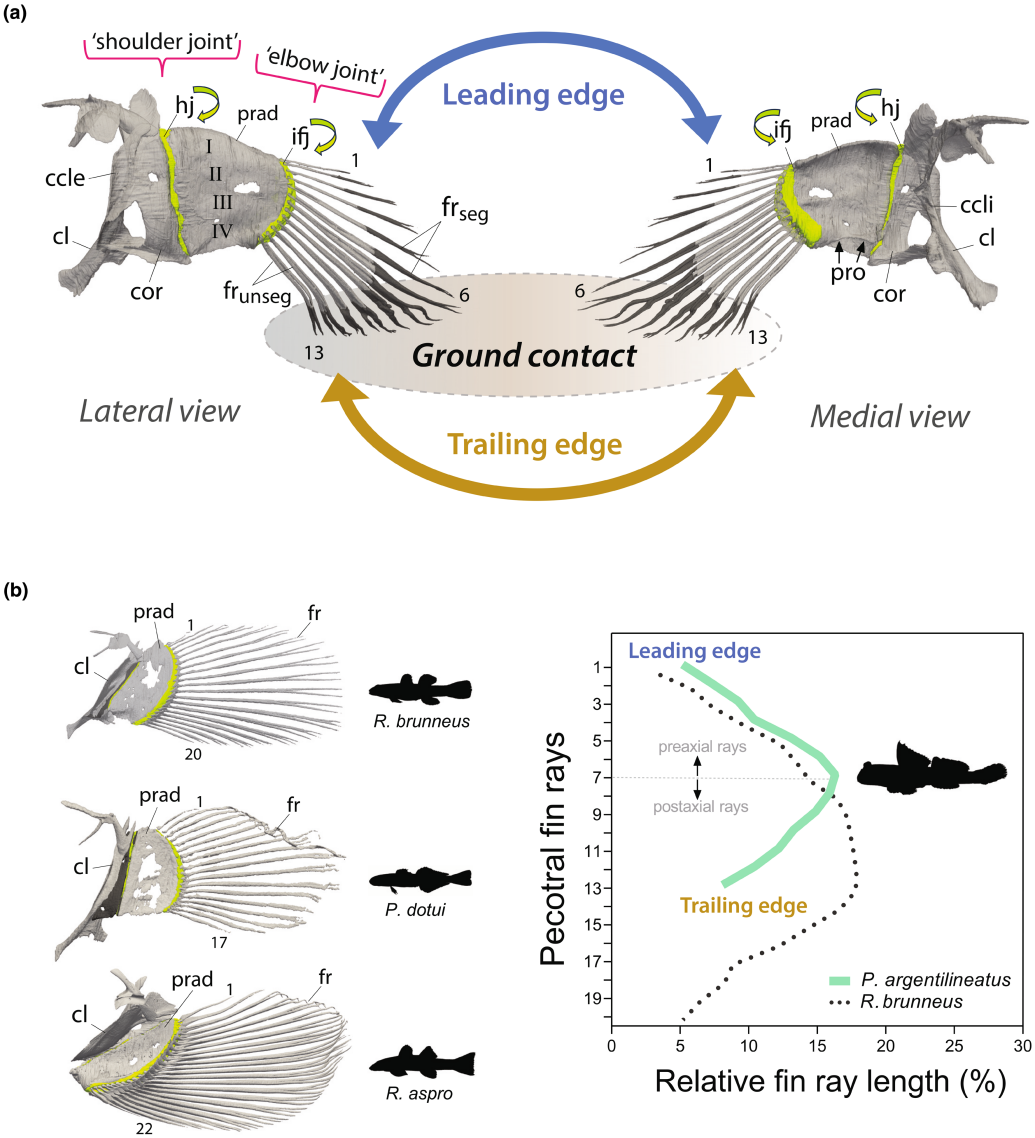
(a) Anatomical structure of the mudskipper's pectoral fin, highlighting the key skeletal elements and points of ground contact for fin rays on the trailing edge, viewed from both lateral and medial perspectives. Neon curved arrows mark the articulating joints. (b) Pectoral fin shape variation in gobies. Fins are in lateral view. ccle, crista cleithralis externa; ccli, crista cleithralis interna; cl, cleithrum; cor, coracoid; fr_seg_, segmented fin ray; fr_unseg_, unsegmented fin rays; hj, hinge joint; ifj, intra‐fin joint; prad, proximal radials; pro, process.

**FIGURE 4:**
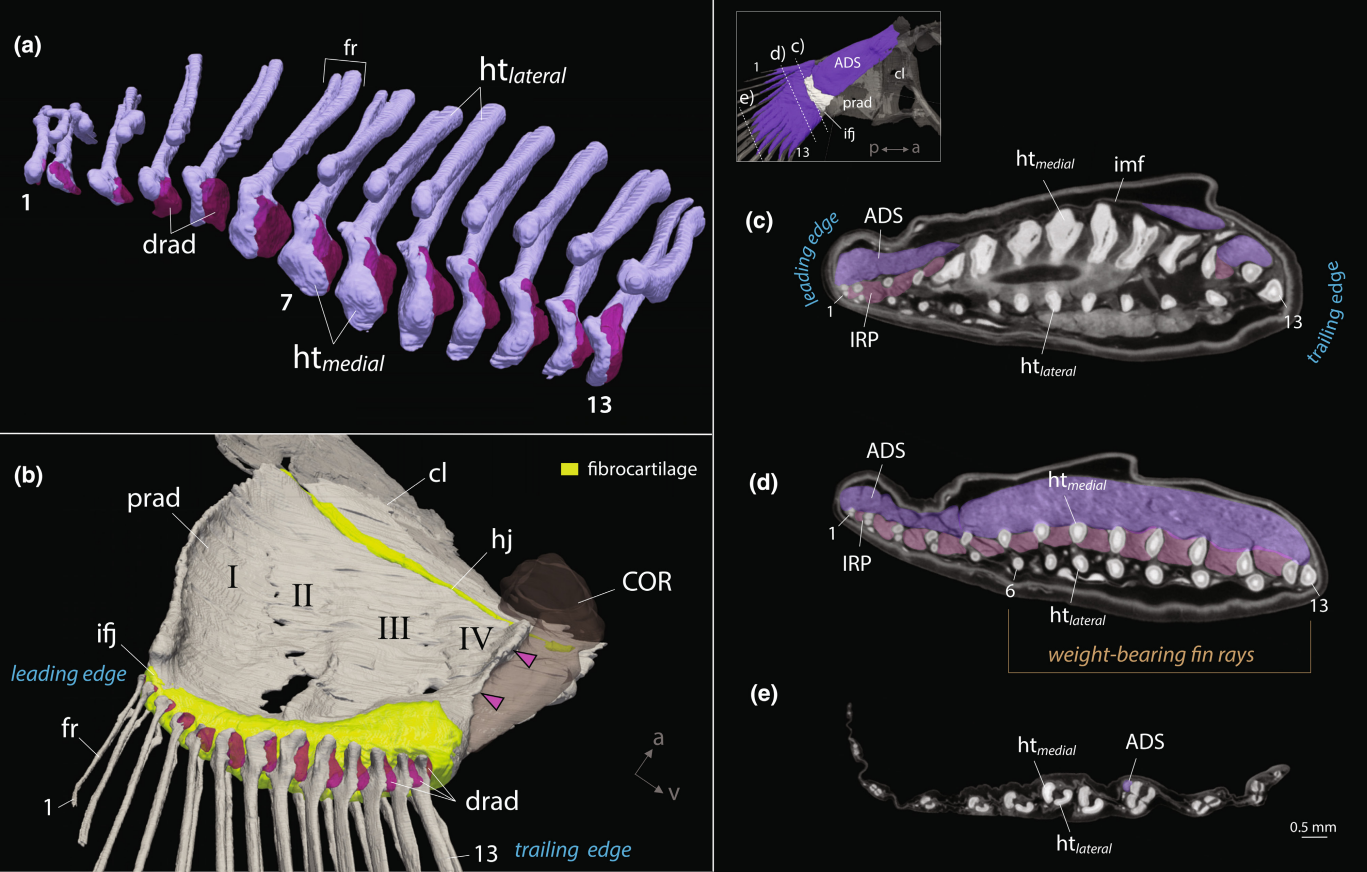
Pectoral fin anatomy of *Periophthalmus argentilineatus*. (a) 3D rendering of pectoral fin rays (fr) at their base. Each fin ray consists of two halves: a lateral hemitrich (htlateral) and a medial hemitrich (htmedial). Thirteen distal radials (drad) are fused to the processes at the base of medial hemitrichs. (b) Volume rendering of the pectoral fin medially. Note the concave surface formed by the elongated proximal radials (prad, I–IV). A process protrudes along the proximal radial IV (pink arrowhead) that separates the coracoradialis muscle (COR). Proximally, the shoulder (cl, cleithrum) articulates with the proximal radials (prad) over a cartilaginous hinge joint (hj). Distally, the fin rays (fr) articulate with the proximal radialis over a cartilaginous pad that forms a flexible intra‐fin joint (ifj). (c–e) Cross‐section slices of μCT of fin rays at (c) the intra‐fin joint (ifj), (d) proximally in the unsegmented region of fin rays, and (e) distally in the segmented region of fin rays (see inset). The structures in (a+b) are rotated ventrally for illustrative purposes. Pectoral fin rays are numbered from dorsal to ventral with the leading ray as ray 1. ADS, adductor superficialis; IRP, interradialis pectoralis; imf, intermuscular fascia; ht, hemitrich.

The base of the pectoral fin consists of four elongated and highly sclerotized basal or proximal radials (I–IV), which decrease in length along the dorsal‐ventral axis (Figures 2b and 3a). This contrasts with other gobies, which exhibit overall shorter proximal radials, culminating in a pectoral fin base that is flat, rounded, and plate‐like (Figure 3b). In mudskippers, the elongated conformation of the proximal radials leads to the pectoral fin's axis declining below the horizontal when the fin is naturally retracted (i.e., pressed against the side of the body), causing the fin to project ventrally (Figures 2 and 3a). The pectoral fin base features a prominently concave surface medially to accommodate the adductor muscles (Figures 3a and 4b). A process protrudes along the fin axis on the medial surface of the ventral‐most proximal radial (IV) (Figures 3a and 4b). The coracoradialis muscle is embedded in the ventral groove of this process (Figure 4b).

The elongated proximal radial bones articulate with the shoulder girdle through a vertically oriented, hinge‐like joint (Figures 2b and 3a). The distal part of the pectoral fin is characterized by 13 webbed fin rays (Figures 2b and 3a). Each fin ray consists of two halves (hemitrichs), which splay at the base and articulate with the radial bones through a substantial, fibrocartilaginous pad (Figures 2b, 3a and 4b). This articulation forms the second joint of the pectoral fin, termed the intra‐fin joint or “elbow‐joint” (Figure 3a). There are 13 smaller distal radials that fuse to the bases of the medial hemitrichs, creating large, bulky processes for muscle insertion (Figure 4a,b). The size and shape of these processes vary along the dorsoventral axis of the fin, with particularly bulky formations occurring in the midline (Figure 4a,c). However, the heads of the lateral hemitrichs are notably smaller. They maintain a consistent roundish shape and lack a process at their base (Figure 4a). In contrast, aquatic gobies feature generally flat bases at the medial hemitrichs and bases with protruding processes on the lateral hemitrichs for tendon attachments (Figure 3b; Figure S2).

In *P. argentilineatus*, the pectoral fin rays are unsegmented for approximately one‐third of their total length proximally and display branching at their distal ends (Figure 3a). The tips of the fin rays are composed of actinotrichia collagens (Durán et al., 2011) (Figure 2b).

**FIGURE 5:**
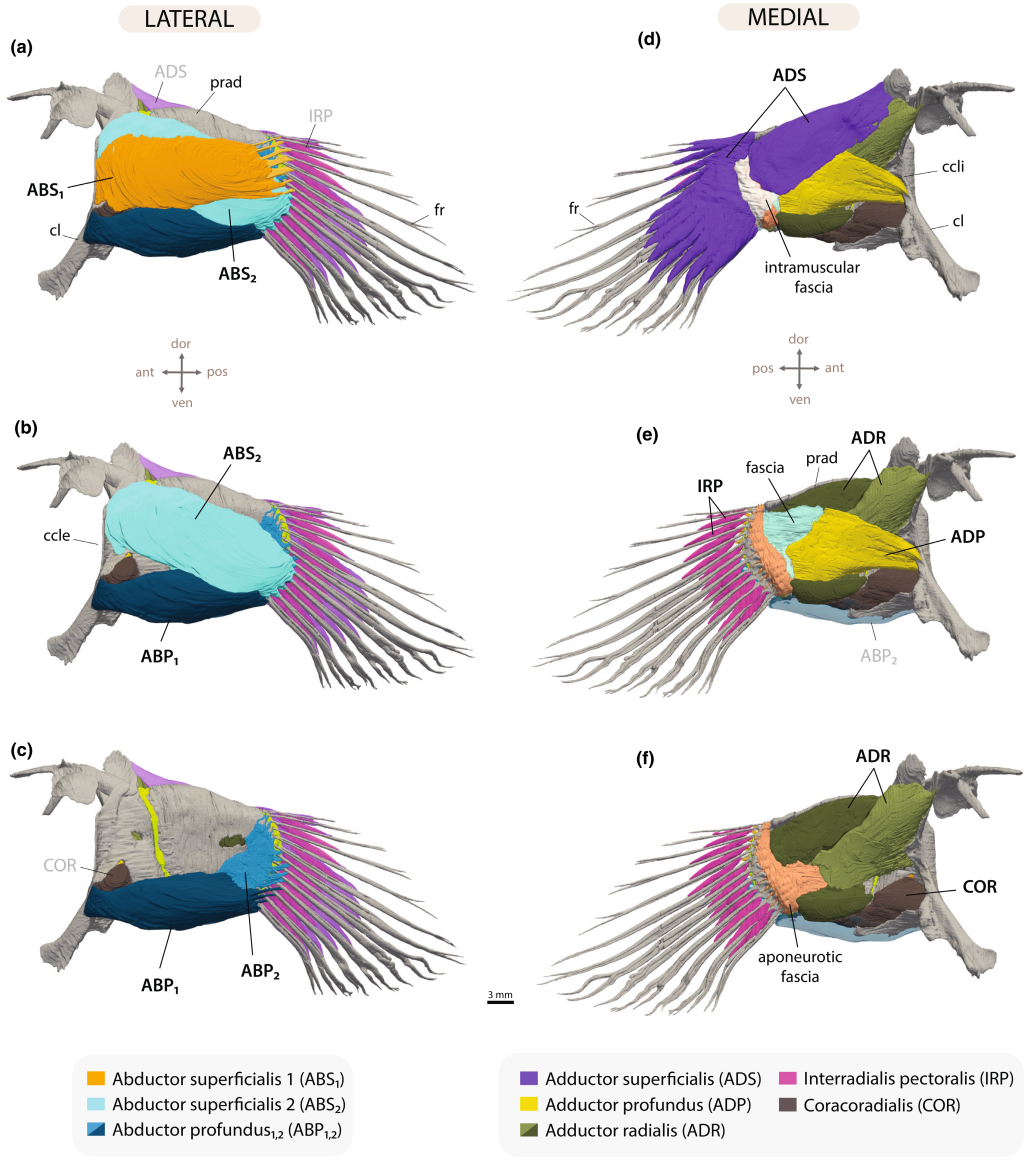
Pectoral fin muscles in *Periophthalmus argentilineatus*. (a–c) Lateral view showing the abductor muscles. (d–f) Medial view showing the adductor muscles and associated fasciae. Muscles are step‐wise removed from top to bottom panel. ccle, christa cleithralis externa; ccli, christa cleithralis interna; cl: cleithrum, fr, fin ray; prad, proximal radials.

The points of attachment for the adductor superficialis (ADS) to the fin rays coincide with the unsegmented portions of these rays (Figure 5d). Branching in the fin rays is particularly evident in those positioned postaxially (Figure 3a). The uppermost five fin rays are comparatively shorter and thinner, likely not making contact with the ground during terrestrial movements (Figure 3a). Conversely, the lower eight fin rays are longer, with those at the fin axis being the longest (Figure 3a). These fin rays are primarily responsible for bearing weight during terrestrial locomotion, particularly when the pectoral fins are fully depressed, and the body's weight is distributed across the lower portion of the fin. This variation in fin ray length contributes to a distinct asymmetric pectoral fin web in *P. argentilineatus* (Figure 3b).

In their proximal unsegmented region, the hemitrichs of *P. argentilineatus* are almost spherical or elliptical in cross‐section (Figure 4d), markedly differing from those seen in aquatic gobies (Figure S1). In addition, the ventral eight fin rays feature larger ossified cores (fin rays 6–13, Figure 4d), aligning with their weight‐carrying function (Figure 3a). Toward the distal end, the hemitrichs gradually transition to a crescent shape (Figure 4e). Hemitrichs in other gobies maintain a consistent crescent shape throughout (Figure S1) and exhibit a more symmetrically formed fin web (Figure 3b). However, *R. aspro* demonstrates a notable thickening in the proximal hemitrichs, particularly evident in the medial hemitrichs (Figure S1a).

#### 3.1.2 Muscle morphology

##### Abductor superficialis 1

**FIGURE 6:**
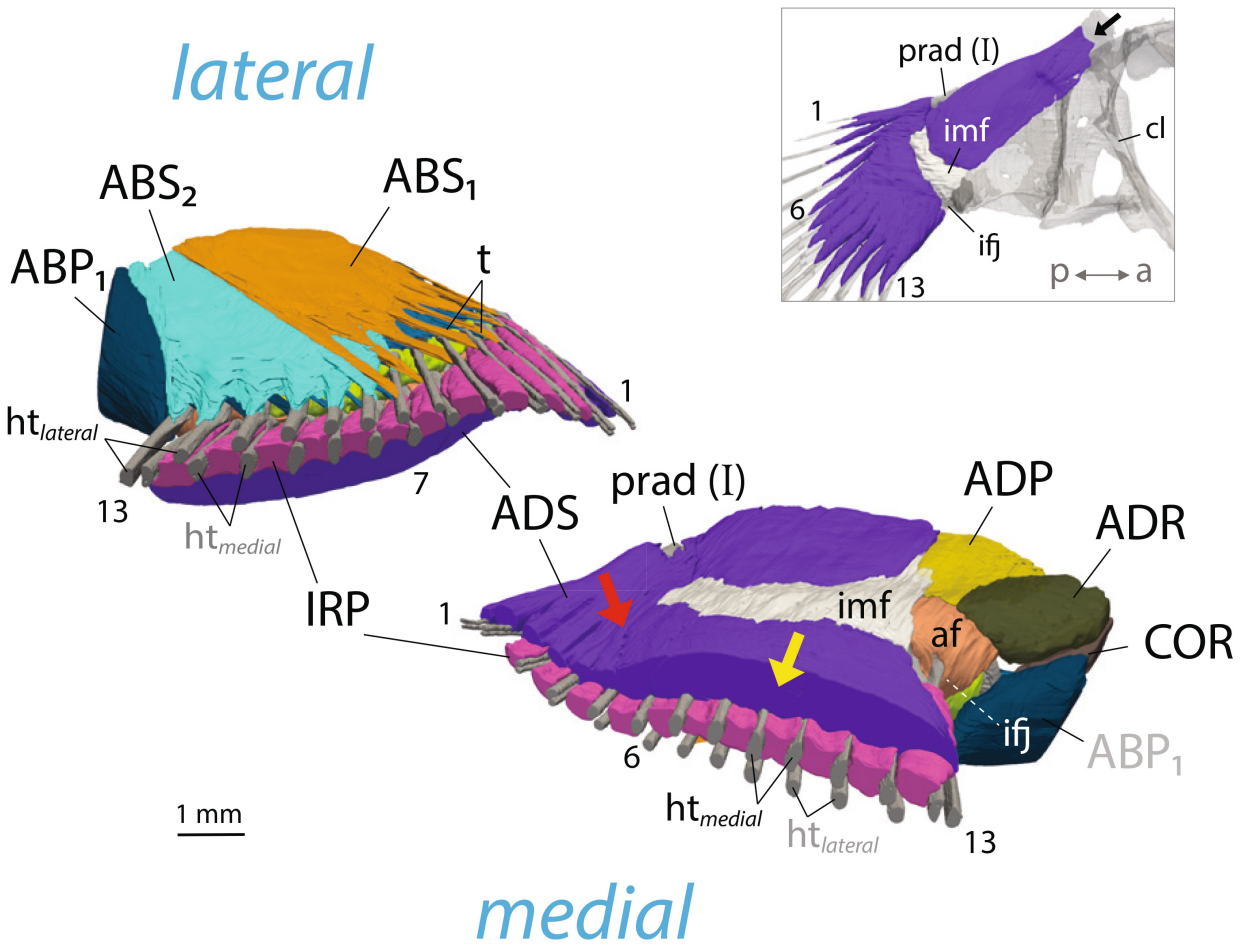
3D renderings reveal specialized superficial muscle formations in *Periophthalmus argentilineatus*'s pectoral fin, highlighted from oblique angles for clarity. Laterally, the superficial abductor mass is divided into two distinct muscles (ABS_1_, ABS_2_). The ABS_1_ (orange) is layered above the ABS_2_ (turquoise) and attaches to the upper fin rays (1–7) through long tendons (t). The ABS_2_ is associated with the lower fin rays (7–13). Medially, the adductor superficialis (ADS, purple) is compartmentalized, having dual origins. The predominant part of the ADS originates dorsally on the cleithrum (black arrow in inset) and diagonally spans to the lower fin rays 6–13 at the trailing edge. The ADS is intersected by an intramuscular fascia (imf, white) at the level of the intra‐fin joint (ifj). Distal to the intramuscular fascia, the ADS develops into a thick muscle layer (yellow arrow) that eventually segregates into individual bundles to directly insert onto the medial hemitrichs (htmedial). The smaller portion of the ADS arises on the pectoral fin base on proximal radial I (prad) at its dorsal edge, with distinct muscle bundles attaching to the upper five fin rays at the leading edge (1–5). Fibers merge at the junction of both ADS muscle compartments (red arrow). Pectoral fin rays are numbered from dorsal to ventral, with the leading ray designated as ray 1. For muscle abbreviation, refer to Table 2. af, aponeurotic fascia; cl, cleithrum; ht, hemitrich; ifj, intra‐fin joint; imf, intramuscular fascia; prad, proximal radial; t, tendon.

In *P. argentilineatus*, the outermost muscle layer segregates into two distinct muscle masses (Figure 5a), whereas in aquatic gobies, this layer still consists of a single superficial muscle mass (green muscle in Figure S2). Positioned most laterally on the pectoral fin, the abductor superficialis 1 (ABS_1_) originates from the middle part of the crista cleithralis externa (Figure 5a). This flat and band‐like muscle accommodates the upper seven fin rays from the leading edge (fin rays: 1–7) (Table 2). The muscle is equipped with elongated tendons that insert latero‐ventrally onto the hemitrichs at some distance from their base, which creates abduction of these fin rays (Figures 5a and 6).

**TABLE 2:** Summary for paired fins and caudal fin muscles in barred mudskipper, *Periophthalmus argentilineatus*.

Fin muscle	Abbr.	Origin	Insertion	Ent.	Function
Pectoral fin					
Abductor superficialis 1	ABS_1_	ccle	fr_lateral_ (1–7)	t	Elevation, abduction
Abductor superficialis 2	ABS_2_	ccle	fr_lateral_ (7–13)	t	Abduction, depression
Abductor profundus 1	ABP_1_	ccle/cor	fr_lateral_ (10–13)	t	Abduction, depression
Abductor profundus 2	ABP_2_	prad II–IV	fr_lateral_ (1–9)	t	Abduction, depression
Adductor superficialis	ADS	cl, prad I	fr_medial_ (cl: 6–13, prad: 1–5)	d	Elevation, adduction
Interradialis pectoralis	IRP	fr (1–12)	adjacent fr below (2–13)	d	Adduction
Adductor profundus	ADP	cl/ccli	af	fa	Adduction
Adductor radialis	ADR	cl, prad I‐IV	fr_medial_ (1–13)	af	Adduction
Coracoradialis	COR	cor, ccle	prad IV	d	Adduction
Pelvic fin					
Abductor superficialis pelvicus	ABSP	ap	fr_ventral_ (1–5), pp	t	Abduction
Abductor profundus pelvicus 1	ABPP_1_	pcar + bas_ventral_	fr_ventral_ (1–5)	t	Abduction
Abductor profundus pelvicus 2	ABPP_2_	pcar	I + fr_ventral_ (1–4)	t	Abduction
Arrector dorsalis pelvicus	ARDP	pcar	I (hook_dorsal_)	t	Protraction, abduction
Arrector ventralis pelvicus	ARVP	pcar	I (hook_ventral_)	t	Protraction, abduction
Adductor superficialis pelvicus	ADSP	pcar + bas_dorsal_	I + fr_dorsal_ (1–5)	t	Adduction, retraction
Adductor profundus pelvicus	ADPP	pcar + bas_dorsal_	fr_dorsal_ (1–5)	t	Adduction, retraction
Extensor proprius	EXP	pcar	fr_dorsal_ (5)	t	Retraction, adduction
Retractor ventralis	RV	pp	fr_medial_ (5)	d	Retraction
Infracarinalis anterior	ICAR.A.	cl	ap + al	d?	Protraction, depression
Caudal fin					
Flexor dorsalis	FD	pu2, nsp, us, hyp (3+4)/5, ep	fr_dorsal_ (1–7)	t	Abduction, lateral flexion
Flexor dorsalis superior	FDS	ccar, nsp, ep	pcr_dorsal_ (1–10)	d	Abduction
Flexor ventralis	FV	pu2, hsp, us, hyp (1+2), phy	fr_ventral_ (8–14)	t	Abduction, lateral flexion
Flexor ventralis inferior	FVI	ccar, pu2, hsp	pcr_ventral_ (1–10)	d	Abduction
Hypochordal longitudinalis	HL	hyp (1+2)	fr_dorsal_ (2 + 3)	t	Adduction, lateral flexion
Interradialis caudalis	IR	fr	fr (1–14) + 2 pcr	d	Adduction
Interhemitrichialis caudalis	IH	ht	opposite ht (1–14)	d	Fin ray stabilization?

*Note*: Details include muscle origin, insertion, type of attachment (enthesis) and inferred function during terrestrial locomotion. Refer to the accompanying video animation (Video S1) for a visual representation.

Abbreviations: af, aponeurotic fascia; al, anterior lip of basipterygium; ap, anterior process (of basipterygium); bas, basipterygium; ccar, caudal cartilage; ccle, crista cleithralis externa; ccli, crista cleithralis interna; cl, cleithrum; cor, coracoid; d, direct; ent, enthesis; ep, epurals; fa, fascia; fr, fin ray; hyp, hypural; hsp, hemal spine; ht, hemitrich; I, pelvic spine; nsp, neural spine; pcar, pelvic cartilage; pcr, procurrent ray; phy, parhypural; pp, posterior process (of basipterygium); pu2, penultimate caudal vertebra; prad, proximal radial; t, tendon; us, urostyle.

In all aquatic gobies, the abductor superficialis muscle uniformly inserts via tendons onto a protruding process at the base of the lateral hemitrichs (Figure S2).

##### Abductor superficialis 2

The abductor superficialis 2 (ABS_2_) has its origin on the middle and upper part of the crista cleithralis externa and is situated beneath the ABS_1_ (Figure 5b). This muscle is broad and flat, extending diagonally to the ventral fin rays (fin rays: 7–13) (Table 2). Notably, both ABS_1_ and ABS_2_ serve fin ray 7 (Figure 6). The tendons of ABS_2_ are short and inserted more laterally at the proximal ends of the hemitrichs (Figure 6).

##### Abductor profundus 1 and 2

The abductor profundus consists of two portions in all studied gobies (Figure 5c; Figure S2). Although each portion originates distinctly on the fin, they do share muscle bundles at the points where the portions overlap. In *P. argentilineatus*, the “superficial” part of the abductor profundus (ABP_1_) is fusiform and voluminous, originating from the lower part of the shoulder girdle, specifically from the crista cleithralis externa and the coracoid (Figure 5c). The muscle bundles of this part extend parallel along the horizontal fin axis and connect to the last four fin rays at the trailing edge (fin rays: 10–13) (Table 2).

The deeper situated portion of the abductor profundus (ABP_2_) is significantly reduced in *P. argentilineatus* compared with its aquatic counterparts (Figure 5c; Figure S2). It consists of a thin layer of muscle fibers originating distally on proximal radials II–IV, fanning out to serve the upper nine fin rays at the leading edge (fin rays: 1–9) (Table 2). The insertions of both the ABP_1_ and ABP_2_ on the fin rays onto the pectoral fin rays are oriented ventrally on the surface of the lateral hemitrichs (Figure 5c). For the upper five fin rays at the leading edge, the tendons are inserted closer to the base. For the consecutive fin rays, the tendons insert at a distance from the base. This arrangement facilitates efficient fin flexion and concurrently strong depression across the ventral part of the pectoral fin during terrestrial locomotion (Table 2).

In aquatic gobies, the ABP_1_ is a flat muscle that originates from the lower to middle portion of the cleithrum (Figure S2). It extends dorsally into the fin plate, overlaying the ABP_2_, which typically arises from all four proximal radials (Figure S2). Both portions of the ABP connect laterally through short tendons to the lateral hemitrichs' surfaces (Figure S2).

##### Adductor superficialis

The adductor superficialis (ADS), referred to as m. levator superficialis in Harris (1960), is the medially situated outermost muscle of the pectoral fin and exhibits a complex structure in *P. argentilineatus* (Figure 5d). Essentially, this muscle is comprised of two marginally interconnected segments, each originating differently on the fin. The more substantial segment of the ADS originates on the cleithrum's dorsal tip, extending long muscle bundles diagonally across the pectoral fin plate to the lower eight fin rays at the trailing edge (fin rays: 6–13) (Figure 6; Table 2). At the intra‐fin joint level, this muscle intersects with a thin layer of connective tissue, herein termed the intramuscular fascia (Figures 5d and 6). After this fascial layer, the ADS proceeds as a dense muscle layer, diverging into separate bundles approximately halfway from the fascia, attaching along the dorsal surface of the medial hemitrichs (Figures 5d and 6). The muscle bundles are markedly thick and long along the fin rays and are specifically designated to support the lower eight fin rays at the trailing edge, which possess larger ossified cores (Figures 4d and 6).

The smaller segment of the ADS consists of a thinner layer of muscle fibers that originate dorsally and distally on the pectoral fin plate on proximal radial I (Figure 6). Individual muscle bundles cross directly over the intra‐fin joint, without intramuscular fascia, and insert onto the medial hemitrichs of the first five fin rays at the leading edge (fin rays: 1–5) (Figures 5d and 6; Table 2). The attachment takes place more dorsally and slightly laterally on the hemitrich surface. At the juncture of both segments of the ADS, several muscle bundles are integrated (Figure 6).

Given the insertion of the ADS onto the hemitrichs is oriented dorsolaterally, activating this muscle elevates the entire array of fin rays (Harris, 1960). Furthermore, the ADS is thought to assist in the fin's maneuverability, such as during rotational movements when one pectoral fin is lifted off the ground and retracted for repositioning. In contrast to *P. argentilineatus*, aquatic gobies do not exhibit a comparable, intricate structure in the ADS. In these species, the ADS is characterized by a relatively uniform muscle mass (Figure S2). This mass originates from the dorsal part of the cleithrum and extends dorsally on the uppermost proximal radial. The muscle bundles of the ADS insert directly onto the dorsal or lateral surface of all fin rays, maintaining some distance from their base (Figure S2).

##### Interradialis pectoralis

In *P. argentilineatus*, adjacent fin rays of the pectoral fins are interconnected by additional muscle bundles, which are absent in aquatic gobies (Figure 5; Figure S2). This group of muscle bundles is referred to as interradialis pectoralis (IRP, sensu m. interradii in Harris (1960)). The IRP, positioned beneath the muscle bundles of the ADS (Winterbottom, 1973), is evidently derived from this muscle group (Figure 6).

The IRP encompasses 12 distinct muscle segments located between the fin rays (Figures 4d and 6; Table 2). Proximally, individual muscle bundles emerge a short distance from the heads of medial hemitrichs. These fibers take an oblique, or diagonal, path toward the adjacent ventral fin ray. They attach to the dorsal side of both hemitrichs in fin rays 1–5 (Figure 4d). However, for fin rays 5–13, they only insert on the medial hemitrich (Figure 4d).

When activated (adducted), the fin rays come together, causing the fin web to fold. Relaxing these muscle segments then allows the fin web to spread out again. The IRP is presumed to play a crucial role in modulating and controlling pectoral fin shape during terrestrial locomotion.

##### Adductor profundus

The deeper muscle mass positioned medially on the pectoral fin, and originating from the lower part of the cleithrum, has been described as adductor profundus (ADP) in mudskipper *Boleophthalmus boddarti* (refer to fig. 12 in Eggert (1929)), in European goby, *Pomatoschistus lozanoi* (fig. 7b,e in Adriaens et al., 1993), and also in zebrafish (Siomava & Diogo, 2018).

In other mudskipper species, the deeper muscle mass of the medial pectoral fin originates from the middle section of the cleithrum. For example, in *Periophthalmus koelreuteri (= barbarus*; Murdy, 1989), this muscle was referred to as the m. extensor medialis (see fig. 8 in Harris, 1960), whereas in *Periophthalmodon schlosseri* and *Periophthalmus chrysospilos*, it was identified as the adductor medialis (refer to figs. 20 and 25, respectively, in Eggert, 1929). However, based on our CT scans from several gobies, we postulate that there is no distinct “medialis” muscle in mudskippers, suggesting instead that this muscle is synonymous with the ADP.

**FIGURE 7:**
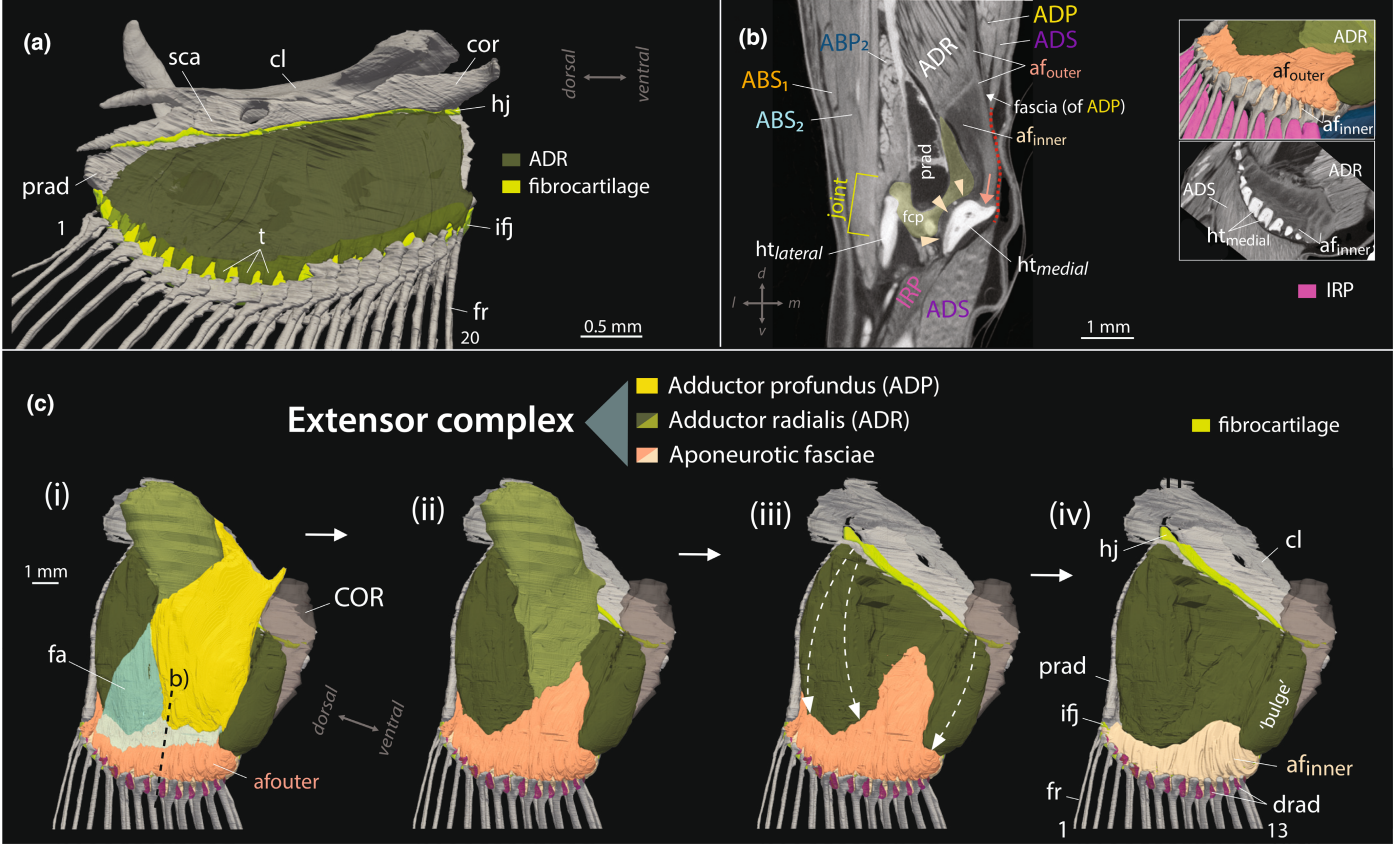
Soft tissue diversification and specializations of the deep adductors of the medial mudskipper pectoral fin. (a) Comparative 3D rendered pectoral fin of the aquatic goby, *Rhinogobius brunneus*, with the deep adductor radialis (ADR, dark green) shown, suggesting homology of the ADR across gobies. (b) Coronal μCT section of the pectoral fin in *Periophthalmus argentilineatus* at the intra‐fin joint reveals two distinct aponeurotic fasciae associated with adductor radialis (ADR). The outer fascia (afouter) is dense and “strap‐ like,” connecting directly to the base of all medial hemitrich (htmedial) heads (arrow, dark rose). In contrast, the inner aponeurotic fascia (afinner) is less fibrous and wraps around the large processes of the medial hemitrich heads (arrowheads, light rose). The inner aponeurotic fascia is positioned atop the fibrocartilaginous pad (fcp) of the joint. The fascia of the adductor profundus (ADP) merges with the outer aponeurotic fascia (white arrow). The dotted red line indicates the intramuscular fascia of the adductor superficialis (ADS), extending over the joint. The insets show an oblique view of both aponeurotic fasciae and a corresponding μCT image in a sagittal slice. (c) 3D‐rendered pectoral fin of *P. argentilineatus*, illustrating the “extensor complex” after the removal of the ADS. This complex integrates the strength of the ADP (ADP, yellow) and the compartmentalized adductor radialis (ADR, light and dark green) through extensive aponeurotic fasciae (light and dark rose). An additional thin fascia layer (fa) connects the ADP to the outer aponeurotic fascia (afouter, dark rose). The superficial segment of the ADR (light green) takes its origin from the shoulder and connects to the outer aponeurotic fascia, while the deeper section of the ADR (dark green) partially attaches to the outer fascia (indicated by white dashed arrows) but primarily to the inner aponeurotic fascia (afinner, light rose). Muscles and connective tissue are progressively removed from left (i) to right (iv). Fins in (a) and (c) have been rotated approximately 90° ventrally for visualization. For muscle abbreviations, see Table 2. af, aponeurotic fascia; cl, cleithrum; cor, coracoid; drad, distal radial; fa, fascia; fcp, fibrocartilaginous pad; fr, fin ray; hj, hinge joint; ht, hemitrichia; ifj, intra‐fin joint; prad, proximal radial; t, tendon; sca, scapula.

In *P. argentilineatus*, the ADP attaches to the middle to lower part of the cleithrum, specifically at the crista cleithralis interna (Figure 5e). A wide fascial sheet connects the ADP to an underlying aponeurotic fascia (Figures 5e and 7c–i). The muscle bundles located dorsally on the crista cleithralis interna serve the upper fin rays at the leading edge, whereas the fibers emerging from deeper within the cleithrum reach the ventral fin rays through the fascial sheet and subsequent aponeurosis (Figure 7c–i; Table 2).

In aquatic gobies, the ADP serves as a substantial muscle, typically encompassing a significant portion of the pectoral fin plate (Figure S2). The ADP adheres to the medial hemitrichs via individual tendons in European gobies (Adriaens et al., 1993), attaching to the protrusions of all these hemitrichs. However, in goby species, such as *R. aspro, R. brunneus* and *P. dotui*, the uppermost fin rays are not included in this attachment (Figure S2). Generally, this muscle originates from the middle and lower sections of the cleithrum and expands to include the coracoid.

##### Adductor radialis

We identify the innermost muscle of the medial pectoral fin in all gobies studied here, including *P. argentilineatus* and aquatic gobies, as the adductor radialis (ADR) (Figures 5f and 7; Figure S2), connecting the proximal radials to the fin rays (Winterbottom, 1973). Notably, the presence of an ADR has not been reported previously in the European goby *P. lozanoi*, nor the mudskipper *B. boddarti* (Adriaens et al., 1993; Eggert, 1929).

Our μCT scans, however, reveal this muscle prominently located on the medial inner surface of the pectoral fin plate. In aquatic gobies, it typically originates from the inner surface of the proximal radials and often extends across the entire inner surface of the fin plate (Figure S2), connecting to the base of all medial hemitrichs through short tendons (Figure 7a). Beyond gobies, the ADR has been documented in surfperch (Drucker & Jensen, 1997), black basses (Borden & Colburn, 2008), and blennies (Zander, 1972), but is absent in zebrafish (Grandel & Schulte‐Merker, 1998; Siomava & Diogo, 2018; Thorsen & Hale, 2005).

In *P. argentilineatus*, the ADR exhibits a robust, voluminous form with a compartmentalized structure (Figures 5f and 7c), originating from both the shoulder and the inner surface of the proximal radials (Table 2). This muscle demonstrates significant interconnection across its extent.

**FIGURE 8:**
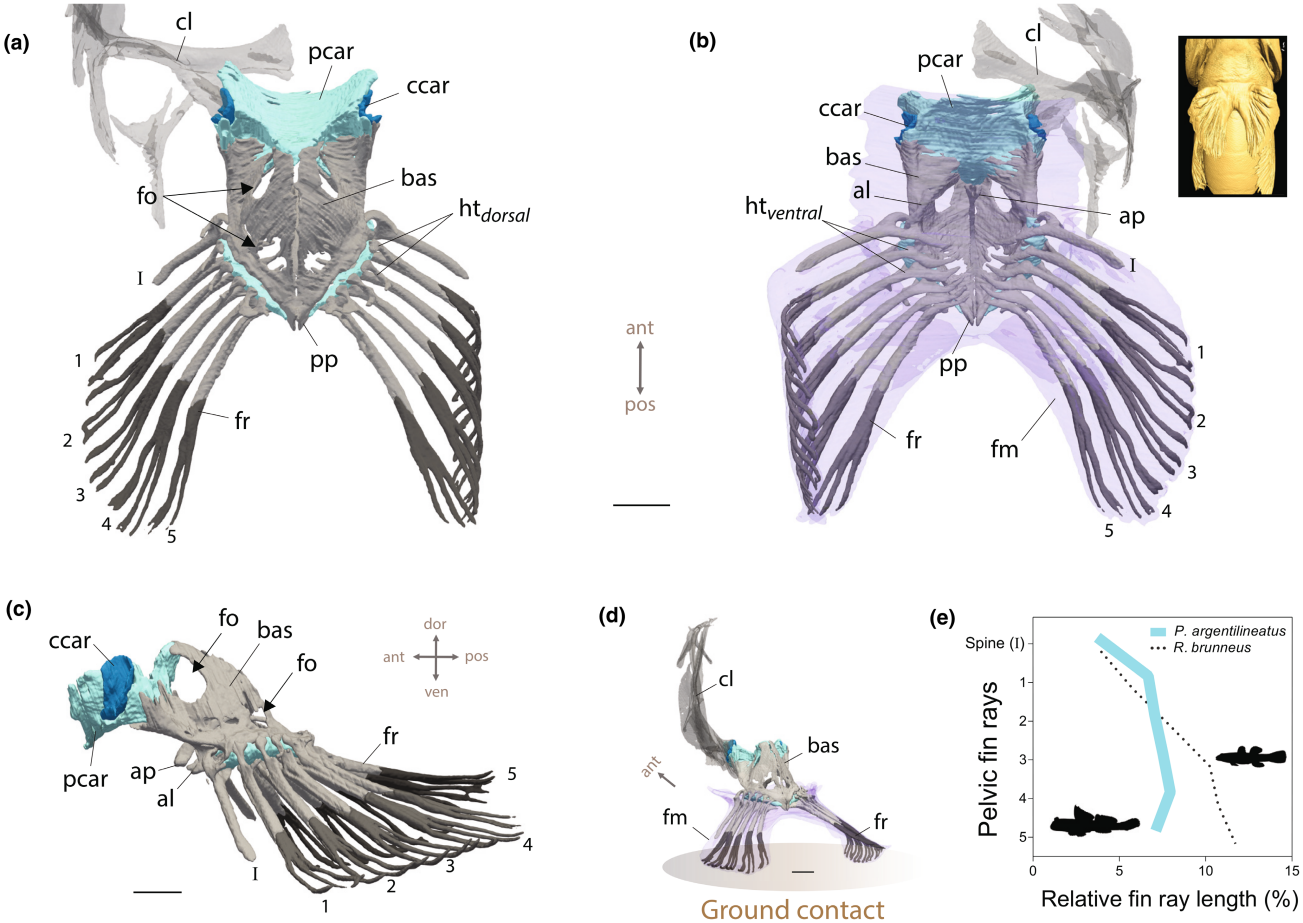
Anatomy of the pelvic fins and girdle in *Periophthalmus argentilineatus*. (a) Dorsal, (b) ventral, (c) lateral, and (d) oblique views. (b+d) *P. argentilineatus* possesses separated pelvic fins with an unfused fin membrane (fm) in the midline. The inset shows an isosurface rendered from a μCT scan, viewed from a ventral perspective. In (c), the left cleithrum is removed to show the joint between the shoulder and pelvic fin. (e) Pelvic fin shape variation in gobies. Segmented fin rays are represented in dark gray and cartilaginous tissue in turquoise. Scale bar: 1 mm. al, anterior lip of basipterygium; ap, anterior process; bas, basipterygium (pelvic plate); ccar, condylar cartilage; cl, cleithrum; fr, fin ray; fm, fin membrane; fo, foramen; ht, hemitrich; pcar, pelvic cartilage; pp, posterior process. I: spine; 1–5: soft fin rays.

The superficial segment of the ADR (sensu m. extensor ventralis in fig. 8, Harris (1960); sensu add. prof. in figs 19 and 25, Eggert (1929)), arises from the dorsal region of the cleithral plate, and to a certain extent, the crista cleithralis interna (light green in Figure 7c). These fibers follow a diagonal trajectory targeting the ventral fin rays and cross over the hinge joint (Figure 7c‐ii).

Beyond the hinge joint, the muscle integrates into an anterior extension of an outer, strap‐like aponeurotic fascia, assuming an “L‐shaped” configuration (Figure 7c‐iii). This fascial band establishes a connection with the heads of all medial hemitrichs (Figure 7b). The more profound segment of the ADR (dark green in Figure 7c) aligns with what Harris (1960) termed as the m. extensor profundus in his fig. 8, and to what Eggert (1929) referred to as add. prof. II in his fig. 20.

In *P. argentilineatus*, this segment of the ADR originates exclusively from the inner surface of the proximal radials and is demarcated proximally by the hinge joint (Figure 7c‐iii). At its most ventral point, the ADR exhibits a pronounced “bulge,” anchoring itself to the hilum of the protruding process on proximal radial IV (Figure 7c‐iv).

The outermost muscle layer of this deeper section of the ADR connects to the outer, strap‐like aponeurotic fascia (white arrows in Figure 7c‐iii). The remaining inner muscle layers attach to the fin rays through a separate inner, and more robust aponeurotic fascia (Figure 7c‐iv). This fascia encases the pronounced processes of all medial hemitrichs (Figure 7b). Although this secondary aponeurotic fascia is thicker, it contains fewer fibrous material compared with the external strap‐like band (Figure 7b). The μCT scans did not determine whether these two aponeurotic fasciae fuse at any point or remain separate.

In *P. argentilineatus*, the ADP, ADR, and their associated aponeurotic fasciae form the “extensor complex” of the pectoral fin (Figure 7c), based on the description by Harris (1960) for a similar mudskipper species. This powerful adductor complex is activated during the power stroke or extension phase, facilitating the fish's forward propulsion over its pectoral fins.

##### Coracoradialis

The coracoradialis (COR) originates from the medial aspects of both the coracoid and the cleithrum, specifically above the condyle (Figure 5f). It inserts into the groove of the prominent process on proximal radial IV (Figure 4b). In *P. argentilineatus*, specific muscle fibers pass through the scapulocoracoid fenestra, attaching laterally to the ventral section of the crista cleithralis externa (Figure 5c; Table 2). In this area, the COR is situated beneath the ABP_1_.

Harris (1960) identified the COR as the only muscle directly serving as an adductor for the pectoral fin plate. This function could be vital for terrestrial locomotion, offering stability to the shoulder joint and the entire fin.

In aquatic gobies, the COR appears as a vestigial muscle, originating from the coracoid and located at the ventral edge of radial IV (Figure S2).

### 3.2 Pelvic fin

#### 3.2.1 Skeletal fin anatomy

The pelvic girdle in *P. argentilineatus* is a dome‐shaped structure composed of two semi‐ossified pelvic bones (basipterygia) that closely approximate each other in the midline, creating a pronounced concavity on the ventral side to accommodate the pelvic abductor muscles (Figure 8; Figure S3d). Each basipterygium features two foramina: the larger foramen is positioned at the anterior end of the pelvic plate (Figure 8a–c), through which the anterior portion of the ventral deep abductor muscle of the pelvic fin (ABPP_1_) attaches and passes (Figure 11a); a smaller foramen is located at the posterior end of the basipterygium (Figure 8a–c).

The lateral edge of the basipterygium in *P. argentilineatus* is ossified and rounded. Cartilaginous padding is present on the lateral region of the basipterygium, offering a movable articulation point for the spine and five soft fin rays (Figure 8). The fin rays exhibit branching and are segmented for about half of their total length, while the spine lacks segmentation (Figure 8a–c).

The pelvic fin rays consist of both dorsal and ventral hemitrichs. The dorsal hemitrichs, along with the spine, form a hook‐like process at their base, creating an insertion point for the dorsal adductor muscles (Figure 8a). Conversely, the ventral hemitrichs – the spine included – extend into a foot‐like shape at their base, serving as an attachment site for the ventral abductor muscles (Figure 8b). All pelvic fin rays exhibit roughly the same thickness and length (Figure 8e). The spine is shorter compared with the fin rays (Figure 8).

In *P. argentilineatus*, the anterior process is situated ventrally and medially on the basipterygium and comprises a thin, elongated, and curved structure extending anteriorly until the level of the pelvic fin spine (Figure 8b,c). The left and right anterior processes fuse at the midline to create a singular structure (Figure 8b). This unified structure acts as an attachment point for both the ventral abductor superficialis pelvicus (ABSP) as well as the infracarinalis anterior (ICAR.A.), the latter of which connects the shoulder to the basipterygium (Figure 11c,e). The posterior process in *P. argentilineatus* is short and pointed (Figure 8a,b).

**FIGURE 9:**
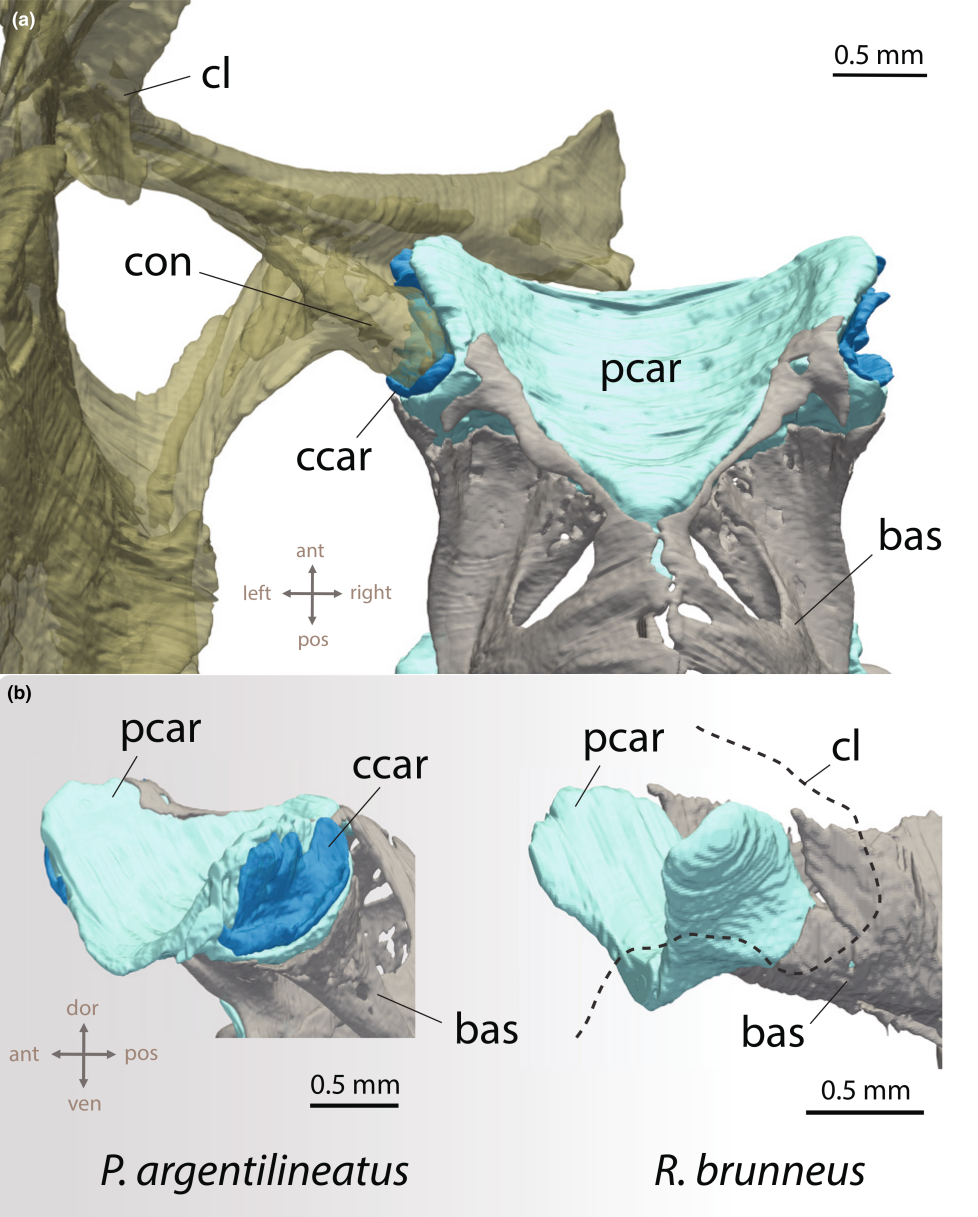
Synergy of shoulder and pelvic girdle in *Periophthalmus argentilineatus*. (a) Top view (the left shoulder is shown). The glenoid joint facilitates the connection between the shoulder condyle (con) and the pelvic cartilage (pcar). A sheet of fibrous material (ccar, condylar cartilage) encapsulates the head of the shoulder condyle. (b) The left image illustrates the joint region and its associated condylar cartilage (ccar) from a lateral, slightly oblique perspective (with the cleithrum removed) in *P. argentilineatus*. In contrast, in aquatic gobies like *Rhinogobius brunneus* (right), the shoulder is loosely connected to the pelvic girdle on the side through a flat extension instead of a condyle. In this configuration, neither a distinct joint nor condylar cartilage is evident. ccar, condylar cartilage; cl, cleithrum; con, condyle; bas, basipterygium; pcar, pelvic cartilage.

**FIGURE 10:**
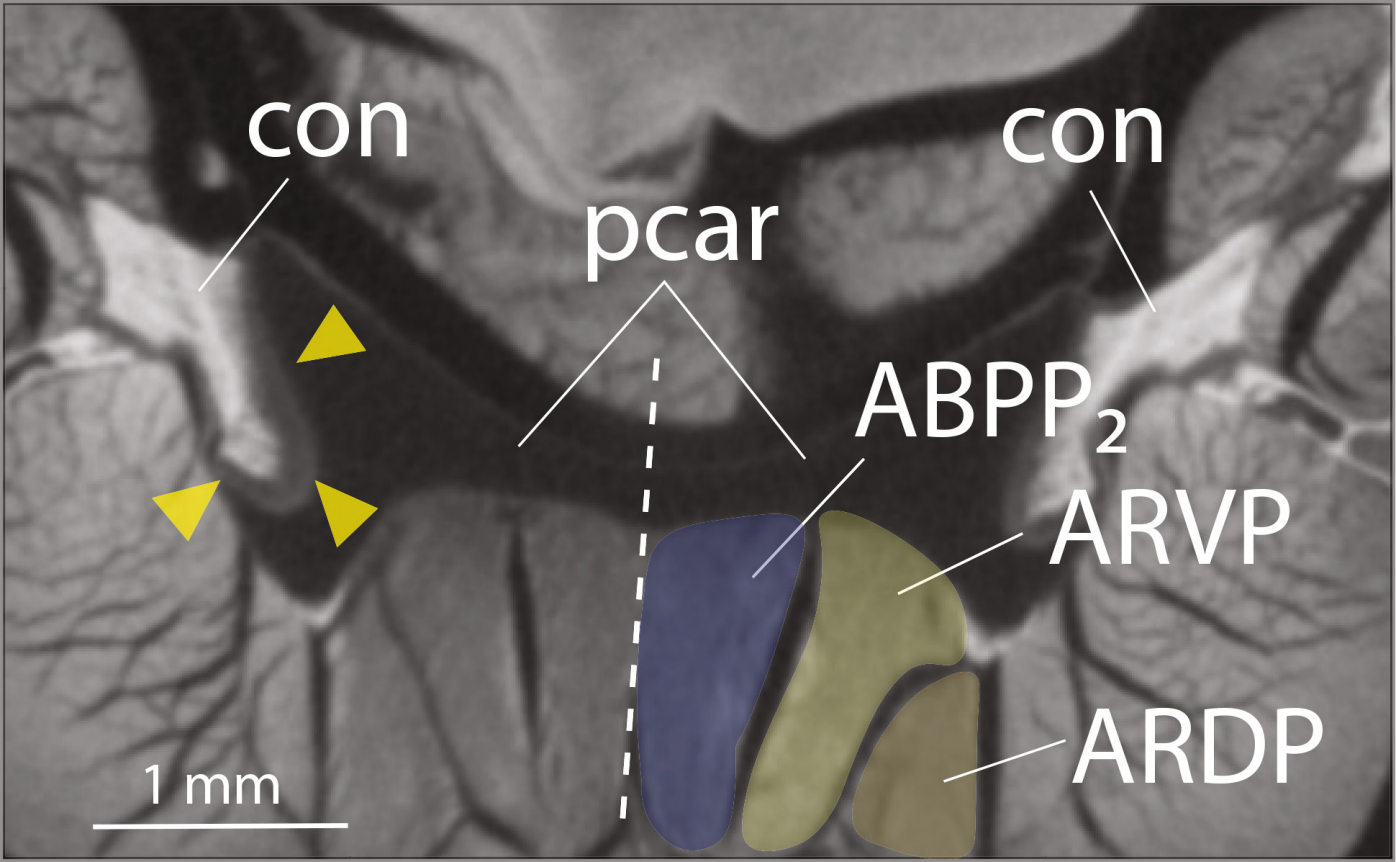
Cross‐sectional view from a μCT scan, highlighting the glenoid joint area between the shoulder and pelvic girdle. Fibrous condylar cartilage (indicated by yellow arrowheads) encompasses the shoulder's condyle (con). The dashed line indicates the body's midline (dorso‐ventral axis). con, condyle of shoulder; pcar, pelvic cartilage; ABPP2, abductor profundus pelvicus 2; ARDP, arrector dorsalis pelvicus; ARVP, arrector ventralis pelvicus.

Anteriorly, the basipterygium merges with a substantial pad of pelvic cartilage (Figures 8, 9, 10). This pelvic cartilage acts as a bridge between the left and right basipterygium and provides a joint surface for the pectoral girdle. In *P. argentilineatus*, the shoulder forms an ovoid condyle ventrally (Figures 8a,b and 9). This condyle is laterally embedded within the pelvic cartilage, thereby creating a glenoid joint (Figure 9a). The μCT scans reveal the presence of fibrous connective tissue (termed “condylar cartilage”) that surrounds and supports the condyle of the shoulder (Figure 10).

Externally, the pelvic fins are covered by a prominent fin membrane, but the innermost pelvic fin rays are not joined by this membrane, resulting in “unfused pelvic fins” in *P. argentilineatus* (Figure 8b, d).

The morphology of the basipterygium in aquatic gobies exhibits distinct features when compared with *P. argentilineatus* (Figure S3). For example, the pelvic girdle of *R. aspro* is a wide, robust, and heavily ossified structure with minimal concavity (Figure S3a), while the pelvic bones in *R. brunneus* converge to form a ridge‐like structure, with *R. brunneus*'s pelvic fin acting as a suction disc (Figure S3b). Conversely, *P. dotui* features slender and flat basipterygia (Figure S3c). As a pelagic swimmer, this goby displays a notably reduced pelvic fin structure, including the loss of a pair of fin rays, resulting in four instead of the typical five (Figure S3c). Moreover, in *P. argentilineatus* and *R. aspro*, the pelvic fin rays are aligned along the thickened lateral edges of each basipterygium, placing them more toward the sides (Figure S3a,d). This arrangement contrasts with species like *R. brunneus* and *P. dotui*, where the fin rays cluster toward the posterior end of each basipterygium (Figure S3b,c). These anatomical variations underscore the evolutionary adaptations to different environmental pressures and roles, leading to the observed diversity in goby pelvic fin morphologies.

#### 3.2.2 Muscle morphology

##### Abductor superficialis pelvicus

**FIGURE 11:**
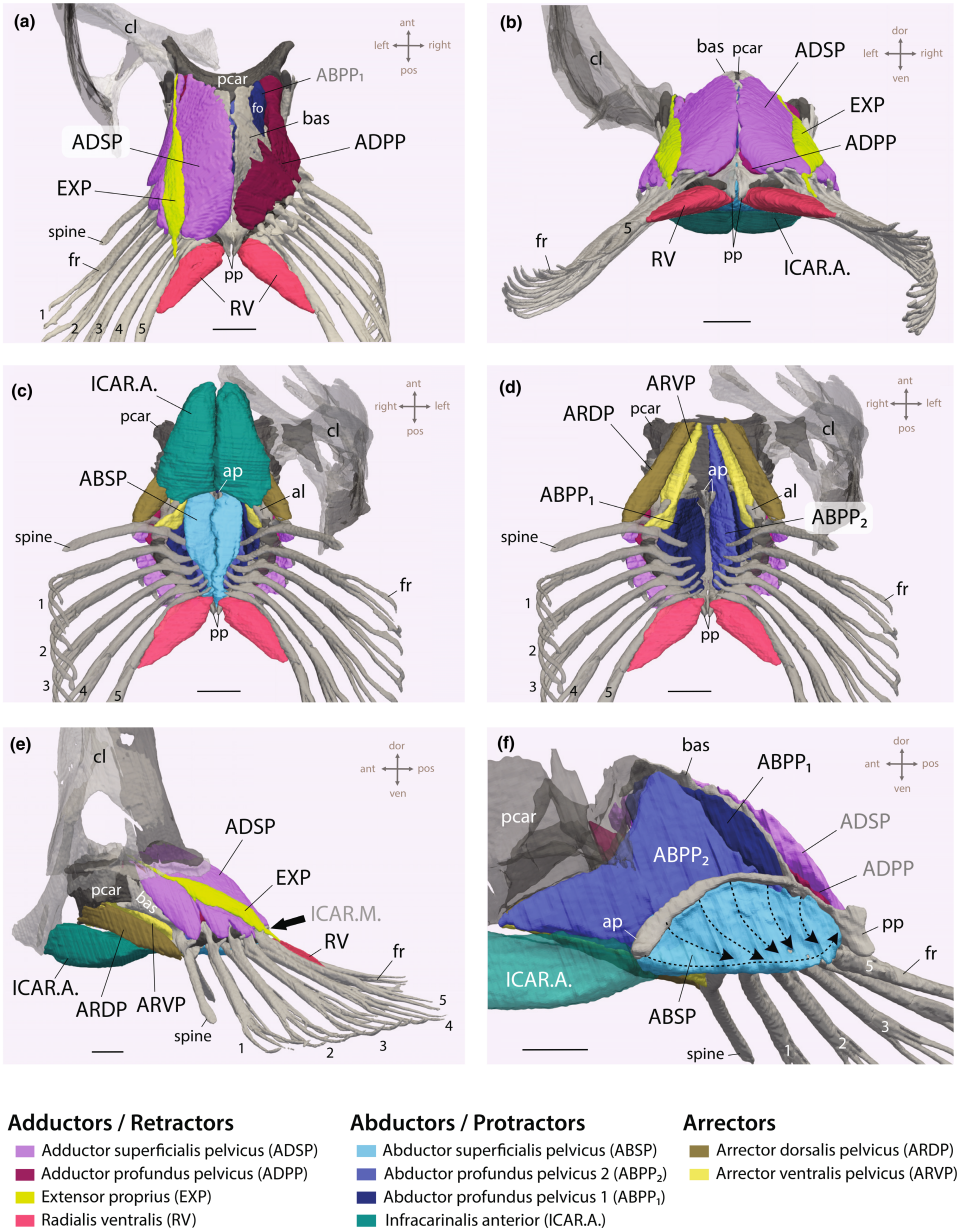
Pelvic fin musculature of *Periophthalmus argentilineatus*. (a) Dorsal view; the superficial muscles, such as the adductor superficialis (ADSP) and extensor proprius (EXP), are removed from the right pelvic fin to reveal the deep adductor muscle (ADPP). (b) Posterior view. (c) Ventral view. (d) Ventral view; the protractor infracarinalis anterior (ICAR.A.), the abductor superficialis profundus (ABSP), and the abductor profundus pelvicus 2 (ABPP_2_) of the right pelvic fin have been removed to expose the ABPP_1_. (e) Lateral view. (f) Lateral view in the midsagittal plane with the left pelvic fin removed. Arrows indicate the direction of fibers for the abductor superficialis pelvicus (ABSP) of the right pelvis. Scale bar: 1 mm. al, anterior lip of basipterygium; ap, anterior process; cl, cleithrum; bas, basipterygium; fo, foramen; fr, fin ray; pcar, pelvic cartilage; pp, posterior process.

The abductor superficialis pelvicus (ABSP) is the outermost muscle on the ventral portion of the pelvic girdle (Figure 11c). This muscle is cone‐shaped, and its attachment spans the entire length of the anterior process of the basipterygium, connecting ventrally and medially (Figure 11c).

The anterior‐most fibers, stemming from the anterior process, extend ventrally and posteriorly, aligning parallel to the fin axis (illustrated by the horizontal dashed line in Figure 11f). These fibers attach not to any fin rays, but to the posterior process. Subsequent muscle bundles arising from the anterior process project almost vertically, attaching to the terminal tips of the processes of every ventral hemitrich (illustrated by vertically oriented dashed lines in Figure 11f). The spine does not receive any fibers from the ABSP. Given the configuration of the muscle bundles and their tendinous connections to the processes, they facilitate the abduction of the fin rays (Table 2).

In aquatic gobies, the ABSP similarly attaches along the anterior process and typically serves all fin rays, though in certain species, like *R. aspro*, it may only serve a few (Figure S3). In *P. dotui*, the anterior process is significantly reduced, allowing the ABSP to extend anteriorly and attach directly onto the pelvic cartilage (Figure S3). While the ABSP usually presents a cone shape, in *R. aspro*, it is flattened (Figure S3).

##### Abductor profundus pelvicus 1 and 2

The deeper portion of the ventral abductors comprises two muscles and rests within the dome‐shaped cavity of the pelvic girdle (Figure 11d). The more internal segment is termed the abductor profundus pelvicus 1 (ABPP_1_). This segment exhibits a somewhat flattened muscle mass, taking origin from the internal wall of the dome (Figure 11d,f). Some fibers also arise dorsally from the cartilaginous pad of the basipterygium, situated just beneath where the superficial adductor muscle (ADSP) attaches (Figure 11a). These fibers navigate posteriorly through the prominent anterior foramen of the basipterygium (Figure 11a). All five pelvic fin rays are served by the ABPP_1_, with the muscle attaching to the base of the processes of all ventral hemitrichs via tendons (Table 2).

The second component of the deep abductor muscle, known as the abductor profundus pelvicus 2 (ABPP_2_), is located medially to the ABPP_1_ in *P. argentilineatus* (Figure 11d,f).

This muscle, which has a flat, vertical layout, originates from the pelvic cartilage and is the innermost muscle in this area (Figure 10). Anteriorly, it attaches vertically (dorsoventrally) to the cartilaginous pad, forming a “V‐shaped” profile when viewed in the midsagittal plane (Figure 11f). The ABPP_2_ connects near the tip of the ventral spine process and to the first four ventral hemitrichia tips, just below the ABSP's insertion point (fin rays 1–4, Table 2). The innermost fin ray does not receive input from the ABPP_2_.

*In P. argentilineatus*, the deep abductors represent the most voluminous muscles of the pelvic fin (Figure S5). The collective activity of both ABPP segments facilitates the downward and outward movement of the fin rays from the body's midline, corresponding to abduction (Table 2).

In aquatic gobies, the ABPP_1_ similarly originates from the internal wall of the basipterygium, and in some cases, from the pelvic cartilage. This muscle usually serves all fin rays. Typically, the muscle is flat and reduced; however, in *R. aspro*, it has developed into a significantly more voluminous form (Figure S3). The ABPP_1_ attaches to the posterior process and extends to the dorsal surface, giving it a “bulging” appearance in *R. aspro* (Figure S3).

The ABPP_2_, positioned medially as the innermost muscle, originates from the pelvic cartilage in aquatic gobies (Figure S3). In *R. brunneus*, it serves only the spine, while in *R. aspro*, it reaches the first two fin rays. This muscle is absent in *P. dotui* (Figure S3).

##### Adductor superficialis pelvicus

The adductor superficialis pelvicus (ADSP) resides on the dorsal surface of the pelvic girdle in *P. argentilineatus*, covering the entire dorsal aspect of the pelvic dome (Figure 11a,b). It originates from the dome's midline, with the anterior portion attaching directly to the pelvic cartilage (Figure 11a). Muscle bundles extend laterally, connecting to the spine and all five fin rays at the uncinate process of each dorsal hemitrich (Figure 11e; Table 2). The ADSP facilitates adduction (movement of the fin rays toward the body's midline) and retraction (backward movement of the fin rays) during terrestrial locomotion, as noted by Harris (1960) (Table 2).

In aquatic gobies, the ADSP has a similar origin and serves all fin rays, including the spine. It is significant in *R. brunneus*, particularly voluminous in *R. aspro*, and appears as a small, band‐like structure in *P. dotui* (Figure S3).

##### Adductor profundus pelvicus

The adductor profundus pelvicus (ADPP) presents as a thinner muscle layer situated beneath the superficial adductor muscle on the basipterygium (Figure 11a). Like the ADSP, the anterior part of the ADPP reaches toward the pelvic cartilage (Figure 11a; Table 2). The remaining fibers originate laterally and further from the midline on the dome, attaching to the base of each dorsal hemitrich via tendinous connections, excluding the spine (Figure 11a; Table 2). During terrestrial locomotion, the ADPP serves a function similar to the superficial muscle, facilitating the adduction and retraction of the fin rays (Table 2).

In aquatic gobies, the ADPP has a similar origin to that in *P. argentilineatus*, yet there is variation in the number of fin rays it serves. *R. aspro* is a distinct case where the ADPP is reduced, positioned at the posterior of the basipterygium, and limited to serving only the innermost fin ray (Figure S3).

##### Extensor proprius

The extensor proprius (EXP) is a superficial, strap‐like muscle situated atop the ADSP in *P. argentilineatus* (Figure 11a,b,e). It features an elongated tendon at its anterior end, originating from the lateral section of the pelvic cartilage (Figure 11a,e). This muscle follows a slightly diagonal path toward the dorsal hemitrich of the fifth and innermost fin ray, attaching to its dorsal surface a certain distance from the base (Figure 11a; Table 2). According to Harris (1960), the EXP slightly twists the last fin ray upward and outward during the pelvic fin's retraction in *P. argentilineatus*.

The EXP in *R. aspro* closely resembles that in *P. argentilineatus* but takes on a distinct form in *R. brunneus* (Figure S3). In *R. brunneus*, the EXP is a pronounced muscle that creates a tent‐like structure covering much of the ADSP, and it attaches to a prominent hook on the innermost fin ray (Figure S3). This configuration likely plays a crucial role in detaching the suction disc from the substrate. The EXP is absent in *P. dotui* (Figure S3).

##### Arrector dorsalis pelvicus

In *P. argentilineatus*, the arrector dorsalis pelvicus (ARDP) manifests as an elongated, slightly triangular‐shaped muscle (Figure 11d,e). Its fibers are aligned in parallel and are positioned on the latero‐ventral side of the basipterygium, placing the ARDP at the pelvic cartilage's most lateral point (Figures 10 and 11d,e). The fibers extend posteriorly to insert onto the anterior surface of the spine's dorsal hook (Figure 11d; Table 2).

Functionally, the ARDP assists in the initial protraction of the spine, orienting it more or less horizontally relative to the ground and subsequently causing the entire fin web to spread out. It is hypothesized that ARDP contraction precedes that of its ventral counterpart, the arrector ventralis pelvicus (ARVP, see below).

In *R. brunneus*, the ARDP's origin and insertion on the spine closely resemble those in *P. argentilineatus* (Figure S3). The ARDP in *P. dotui* is markedly thinner and originates from the lateral side of the pelvic cartilage, whereas in *R. aspro*, it covers a larger area, originating dorsally on the pelvic cartilage (Figure S3).

##### Arrector ventralis pelvicus

The arrector ventralis pelvicus (ARVP) in *P. argentilineatus* is a flat, elongated, triangular muscle located on the ventral side of the basipterygium, and positioned between the ARDP and ABPP_2_ (Figures 10 and 11d). This muscle adheres to the pelvic cartilage, with the fibers extending posteriorly to connect with the ventral process of the spine (Figure 11d). As it progresses toward the spine, the muscle gradually tapers. The ARVP's ventral and inward‐oriented attachment to the spine facilitates forward and inward pulling of the spine upon activation (Figure 11d; Table 2). The sequential activation of the ARDP followed by the ARVP leads to the protraction of the fin rays and their inward and downward movement.

In *R. brunneus*, the ARDV closely resembles that of *P. argentilineatus* in terms of its origin and insertion on the spine (Figure S3). In *P. dotui*, the ARVP is notably thinner and arises from the pelvic cartilage's ventral side, while in *R. aspro*, it is significantly enlarged, indicating a variation in muscle specialization (Figure S3).

##### Radialis ventralis

Absent in aquatic gobies, the radialis ventralis (RV) originates from the posterior process of the basipterygium in *P. argentilineatus* (Figure 11; Figure S3). It attaches to the medial surface of the unsegmented proximal region of the innermost pelvic fin ray (Figure 11; Table 2). This muscle adducts, drawing the last fin rays toward the body as the pelvic fins retract during weight‐loading.

The presence of the RV muscle has been confirmed in multiple *Periophthalmus* species through studies by Eggert (1929) and Harris (1960), though it was notably absent in *Boleophthalmus boddarti* as per Eggert (1929).

##### Infracarinalis anterior

The infracarinalis anterior (ICAR.A.) stands out as a robust muscle with fibers aligned in parallel (Figure 11c,e). It originates from the ventral tip of the cleithrum, extending its fibers posteriorly to attach to the anterior lip of the basipterygium (Figure 11c; Table 2). Another point of attachment is at the tip of the anterior process (Figure 11c; Table 2).

Ranking as the second‐largest muscle within the pelvic region of *P. argentilineatus* (Figure S5), the ICAR.A. is crucial for the protraction (forward movement) and depression (downward adjustment) of the pelvic structure during terrestrial locomotion (Harris, 1960) (Table 2). This muscle enhances the stability in both the pelvis and shoulder girdle, especially during weight‐bearing activities.

The ICAR.A. exhibits uniformity across all studied goby species, with its ventral origin at the shoulder and dual points of insertion evident in every species. In *R. aspro*, it is notably flatter.

The infracarinalis medius (ICAR.M.) acts as the antagonist to the ICAR.A., functioning to elevate the pelvic girdle (Winterbottom, 1973). It originates from the first basal pterygiophore of the anal fin and extends anteriorly to attach to the tip of the posterior process (Figure 11e). The ICAR.M. is part of the hypaxial musculature of fishes and was not measured in this study. We have confirmed the presence of the ICAR.M. in all gobies examined, with its insertion observed on the posterior process.

### 3.3 Caudal fin

#### 3.3.1 Skeletal morphology

**FIGURE 12:**
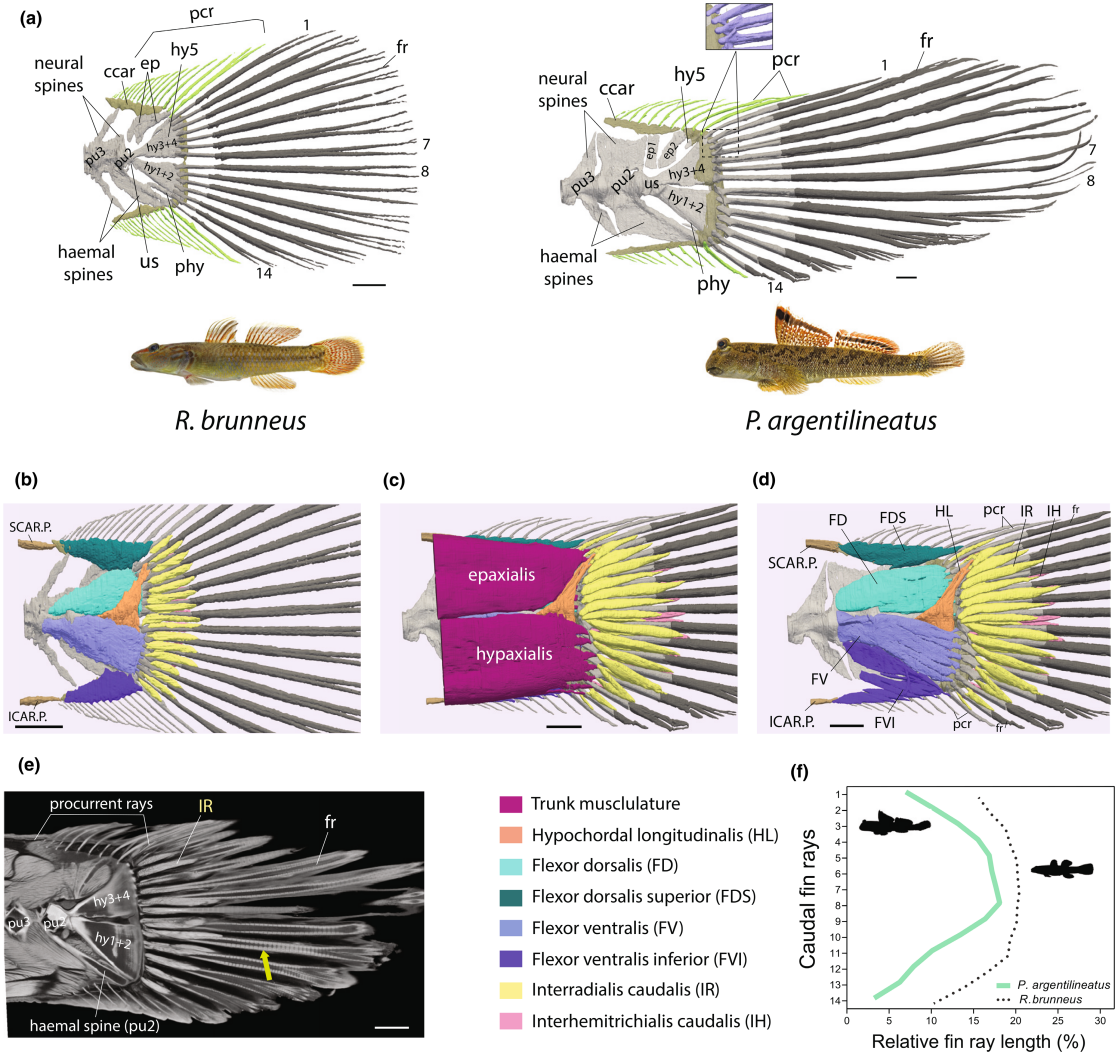
Comparative skeletal morphology of the caudal fin between an aquatic goby (*Rhinogobius brunneus*) and a terrestrial goby (*Periophthalmus argentilineatus*). (a) The caudal fin skeleton in gobies consists of two large hypural plates (hy3+4, hy1+2), an additional dorsal hypural (hy5), a ventral parhypural (phy), and two dorsal epurals (ep1+2). The hemal spine of the penultimate caudal vertebra (pu2) provides support for the most ventral principal caudal fin ray (fr). Principal caudal fin rays are usually branched and exhibit segmentation toward their distal ends (dark gray, green arrow in (e)). *P. argentilineatus* features a reduction in the number of dorsal and ventral procurrent rays (pcr). Rays are supported by cartilage (ccar). The inset shows two magnified hooks on fin rays 2 + 3, which serve as insertion points for the hypochordal longitudinalis (HL) in *P. argentilineatus*. (b–d) In derived teleosts with homocercal (symmetrical) tails, the intrinsic caudal fin musculature typically includes a dorsal flexor (FD) and a ventral flexor (FV), along with the hypochordal longitudinalis (HL) and interradialis caudalis (IR). Procurrent rays align with the dorsal flexor dorsalis superior (FDS) and the ventral flexor ventralis inferior (FVI). Gobies uniquely feature the interhemitrichialis caudalis (IH), which connects the lateral hemitrichia of principal caudal fin rays. The epaxialis and hypaxialis are two superficial layers serving the upper and lower caudal fin rays, respectively, and are extensions of the main body's trunk muscles. (e) A sagittal section derived from a μCT scan showcases the detailed structure of the caudal fin in *P. argentilineatus*. (f) Caudal fin shape variation in gobies. (b) *R. brunneus*; (c–d) *P. argentilineatus*. Scale bar indicates 1 mm. ccar, caudal cartilage; ep, epurals; fr, fin ray; hy, hypural; pcr, procurrent rays; phy, parhypural; pu2, penultimate caudal vertebra; pu3, antepenultimate caudal vertebra; us, urosyle; ICAR.P., infracarinalis posterior; SCAR.P., supracarinalis posterior.

In gobies, the penultimate caudal vertebra (pu2) typically features a short neural spine and an expanded hemal spine, which supports the ventral caudal fin rays (Fujita, 1989) (Figure 12a; Figure S4). In *P. argentilineatus*, both the neural and hemal spines of the pu2 are highly ossified and adopt a plate‐like structure (Figures 2c and 12a). These morphological characteristics increase the attachment area for the caudal fin musculature (Figure 12d). The neural and hemal spines of the antepenultimate caudal vertebra (pu3) are less ossified and closely approximate pu2 (Figure 12a).

At the posterior end of the bony caudal fin in *P. argentilineatus*, two distinct large hypural plates are present, resulting from the fusion of two hypural elements each (specifically, hy1+2 and hy3+4), along with a smaller, independent hypural plate (hy5), a parhypural (phy), and two prominent epural bones (ep1, ep2) (Figure 12a,e).

The caudal fin musculature is supported by a total of 14 segmented, principal caudal fin rays, divided evenly into 7 dorsal and 7 ventral rays (Figure 12a,e). These rays are anchored within a dense cartilaginous pad, drawing support from the hypural plates, the parhypural, and the hemal spine of pu2. The caudal fin exhibits a distinct asymmetrical shape with shorter, “brush‐like” ventral fin rays in *P. argentilineatus* (Figure 12a,f).

The procurrent rays of the caudal fin lack segmentation in both aquatic and terrestrial gobies and are supported by caudal cartilage, accommodating between 10 and 11 dorsal and ventral procurrent rays in *P. argentilineatus* (Figure 12a,e). In addition, the ventral procurrent rays are shorter than the dorsal ones in *P. argentilineatus* (Figure 12a).

While most aquatic gobies display symmetrically shaped caudal fins, species like *R. brunneus* feature a broad and rounded caudal fin, indicative of the typical goby morphology (Figure 12a,f). Conversely, species such as *R. aspro* and *P. dotui* exhibit caudal fins that are emarginate or truncate, respectively (Figure S4).

#### 3.3.2 Muscle morphology

##### Flexor dorsalis

In *P. argentilineatus*, the flexor dorsalis (FD) originates primarily from the penultimate caudal vertebra (pu2), including its neural spine, the urostyle, and the upper hypural bones, with a few fibers also stemming from the epurals (Figure 12d).

This muscle inserts at the base of the upper principal caudal fin rays (1–7) through tendons, facilitating the abduction of these dorsal fin rays (Figure 12d; Table 2). This action expands the surface area of the caudal fin's dorsal lobe, enhancing its flexion and—with regards to swimming—contributing to the propulsion efficiency of the fish (Flammang & Lauder, 2008).

Across all studied gobies, the FD exhibits a consistent structure, origin, and insertion pattern, highlighting a common functional morphology in caudal fin musculature among the species (Figure 12b; Figure S4).

##### Flexor dorsalis superior

The flexor dorsalis superior (FDS) is positioned as the uppermost muscle on the caudal fin of *P. argentilineatus* (Figure 12d). The muscle originates along its entire length from the caudal cartilage, supporting the dorsal procurrent rays (Table 2). The FDS also attaches to the tip of the neural spine of pu2 and the epurals. Its fibers run diagonally to all upper procurrent rays, inserting onto a small protruding hook (Figure 12d). Given the diagonal orientation of the muscle fibers, the FDS is believed to abduct the procurrent rays, aiding in their expansion (Table 2).

In aquatic gobies, the FDS also attaches to the caudal cartilage to support the dorsal procurrent rays (Figure 12b; Figure S4). However, the muscle exhibits species‐specific variation in its connections to neural spines and epurals. For example, in *R. aspro*, the FDS connects to the neural spines of both pu2 and pu3, as well as to the epurals, while in *P. dotui*, it does not attach to these structures at all (Figure S4).

Additionally, in all gobies studied, the fibers of the supracarinalis superior (SCAR.P.) muscle connect the last basal pterygiophore of the dorsal fin to the connective tissue underlying the supporting cartilages of the procurrent fin rays (Figure 12b–d; Figure S4).

##### Flexor ventralis

The flexor ventralis (FV) is positioned ventrally on the caudal fin lobe of *P. argentilineatus*, engaging with the lower principal caudal fin rays 8–14 (Figure 12d; Table 2). It originates from a broad area including pu2 and its hemal spine, the urostyle, the lower hypurals (1+2), and the parhypural, attaching to the base of all seven ventral caudal fin rays through tendons (Figure 12d).

In studies on bluegill sunfish, electrical stimulation of the FV resulted in the abduction of ventral fin rays. This led to an increased surface area of the caudal fin, and consequently, flexion of the ventral lobe, illustrating the muscle's role in swimming efficiency (Flammang & Lauder, 2008).

Across all studied gobies, the FV exhibits a consistent structure, origin, and insertion pattern, highlighting a common functional morphology in caudal fin musculature among the species (Figure 12b; Figure S4).

##### Flexor ventralis inferior

The flexor ventralis inferior (FVI) is situated in the most ventral position on the caudal fin in *P. argentilineatus* (Figure 12d). Its fibers extend anteriorly from the cartilaginous pad of the caudal fin, projecting toward the initial seven ventral procurrent rays (Figure 12d). Additionally, distinct fibers originating from pu2 traverse alongside the hemal spine to serve the last three procurrent rays (Figure 12d). These diagonally oriented muscle fibers insert at some distance from the base of the procurrent rays onto small, protruding hooks (Figure 12d). Similar to its dorsal counterpart, the FVI is believed to abduct the procurrent rays, aiding in their spread due to the diagonal orientation of its muscle fibers (Table 2).

In aquatic gobies, the FVI also originates from and inserts onto the ventral procurrent rays. However, the muscle fibers originating from the hemal spine of pu2 typically do not extend as anteriorly as those in *P. argentilineatus* (Figure 12b; Figure S4).

Additionally, in all gobies studied, the fibers of the infracarinalis posterior (ICAR.P.) muscle connect the last anal fin basal pterygiophore to the connective tissue underlying the supporting cartilages of the procurrent fin rays (Figure 12b–d; Figure S4).

##### Hypochordal longitudinalis

The hypochordal longitudinalis (HL) is the only intrinsic caudal fin muscle that connects ventral and dorsal components of the caudal skeleton, although it is worth noting the potential role of the interradialis caudalis (IR) muscle in this context as well (see below). The fiber orientation occurs at a significant angle to the horizontal axis of the body (Figure 12d). In *P. argentilineatus*, the HL originates broadly from the lower hypurals (1+2) and is partially overlaid by the FV (Figure 12d; Table 2). Distinct fiber bundles extend in a posterior‐dorsal direction to attach to the second and third uppermost fin rays (fin rays: 2+3, Figure 12d), passing over the epaxialis musculature (Figure 12c). The principal caudal fin rays 2 and 3 feature an extended process near their base, serving as the insertion point for the HL's tendons (Figure 12a).

In aquatic gobies, the HL's origin is conserved, with fibers typically attaching to the first two principal caudal fin rays (Figure 12b; Figure S4). Notably, in *R. aspro*, the HL serves the first three fin rays (Figure S4), showcasing species‐specific variations.

The HL muscle plays a pivotal role in the adduction and folding of the upper (dorsal) fin rays, effectively reducing the area of the dorsal lobe, and facilitating lateral flexion. This modulation of the fin rays is critical for teleost locomotion, enabling the generation of lift during consistent swimming patterns. Research by Flammang and Lauder (2009) highlights the HL's contribution to the pronounced lateral movements and higher operational speeds of the dorsal lobe compared with the ventral lobe, underscoring its key role in tail fin modulation.

##### Interradialis caudalis

**FIGURE 13:**
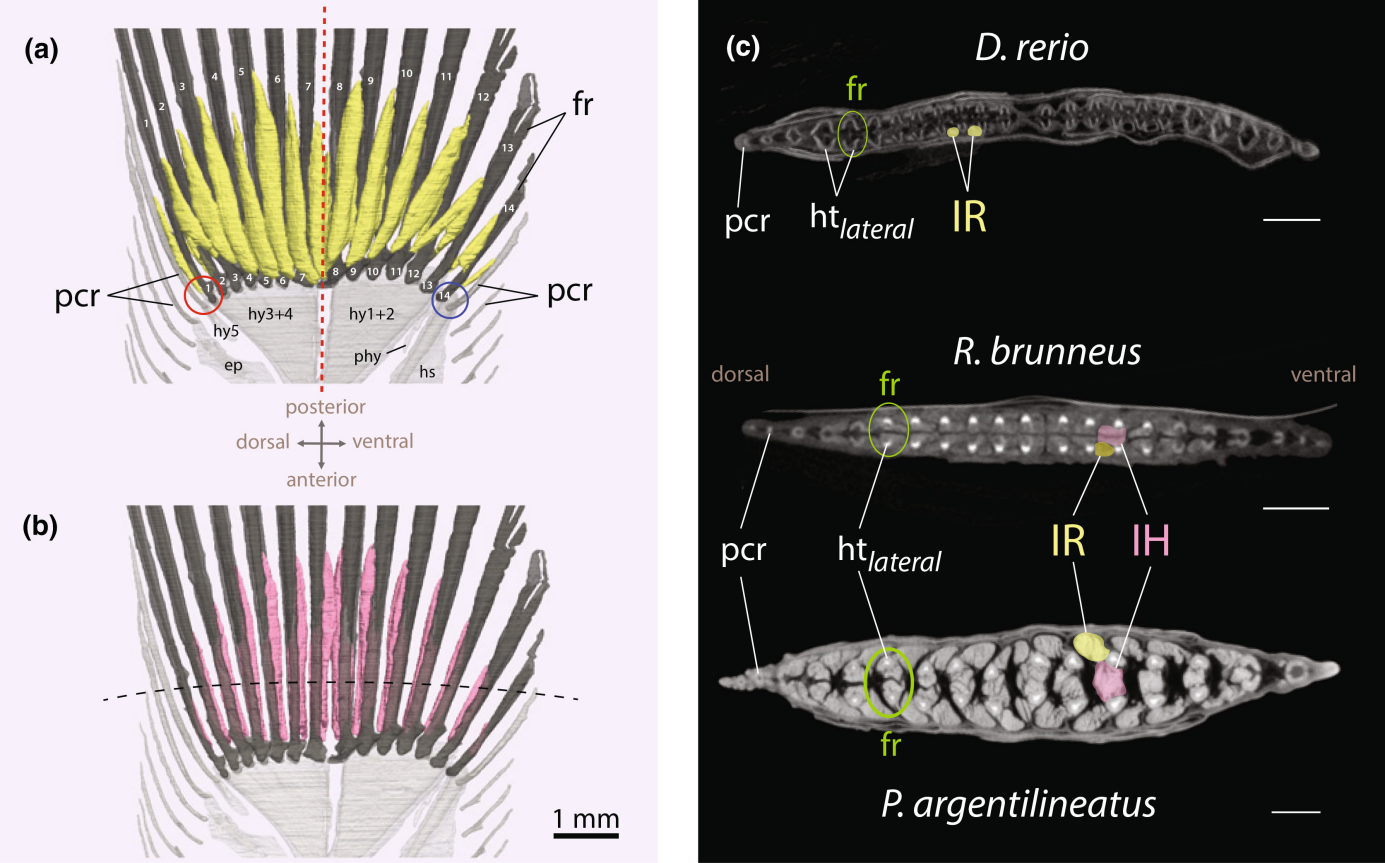
Caudal fin ray musculature in *Periophthalmus argentilineatus*. 3D reconstruction of the (a) interradialis caudalis (IR, yellow) and (b) interradialis hemitrichialis (IH, pink). (c) Cross‐section μCT‐scans showing the principal caudal fin rays (fr, circled in green) at the proximal location (black dashed line in (b)) of zebrafish, *D. rerio*, (upper), *Rhinogobius brunneus* (middle) and *P. argentilineatus* (lower). Muscle bundles that are located between lateral hemitrichia are exclusively found in gobies, but not in zebrafish. Note the difference in muscle thickness of the IH muscles between aquatic and terrestrial gobies. Scale bar indicates 0.5 mm. Red dashed line in (a) indicates the midline of the caudal fin. ep, epural; fr, caudal fin ray; hs, hemal spine; ht, hemitrich; hy, hypural; pcr, procurrent ray; phy, parhypural.

In *P. argentilineatus*, the interradialis caudalis (IR) comprises 15 distinct muscle segments on each side of the caudal fin, positioned directly on the hemitrichs of each principal caudal fin rays (Figures 12d and 13a, c). These segments not only connect the fin rays but also link the first and last principal caudal fin ray (fin rays: 1+14) to their adjacent procurrent ray, originating from a small process at the base of the lateral hemitrichs of each fin ray (Figure 13a; Table 2). In the upper lobe, bundles project upward, inserting onto the ventral aspects of a hemitrich, while in the lower fin lobe, they extend downward to the dorsal aspect.

Starting dorsally at fin ray 1 and progressing down the lobe, the uppermost IR bundle emerges from the first principal caudal fin ray and extends to the adjacent procurrent ray (red circle, Figure 13a), thereby linking the caudal fin ray with the posterior‐most procurrent fin ray. The second IR bundle, connecting to fin ray 1, has a dual origin on fin rays 2 and 3 (Figure 13a). As a result, no bundles from fin ray 3 extend to its immediate neighbor, fin ray 2. The subsequent IR bundles each originate from individual fin rays, stretching to an upper fin ray while skipping its immediate neighbor on the caudal lobe.

Approaching the caudal fin's midline, the IR bundles designated to fin rays 6 and 7 commence both from the base of fin ray 8 in the ventral lobe, crossing the mid‐lateral line (red dashed line, Figure 13a). This arrangement means that in addition to the HL, these two IR bundles emerging from fin ray 8 and targeting fin rays 6 and 7, also serve as connectors, integrating the dorsal and ventral portions of the caudal fin.

In the ventral lobe, fin ray 8 dispatches another two distinct bundles that insert onto fin rays 9 and 10, respectively (Figure 13a). Cumulatively, fin ray 8 serves as an attachment point for a total of four IR muscle bundles. These accommodate fin rays 6 and 7 in the dorsal lobe, along with their ventral counterparts, fin rays 9 and 10. Progressing further, the muscle bundle emerging from fin ray 9 bypasses its immediate neighbor, fin ray 10, to connect with fin ray 11 (Figure 13a). This pattern is consistent for subsequent bundles.

Near the caudal fin's ventral edge, fin ray 13 receives muscle bundles from the preceding fin rays 11 and 12. This arrangement leads to the situation where fin ray 11 does not contribute muscle bundles to its immediate neighbor, fin ray 12. Fin ray 14 directly acquires its muscle bundles from the adjacent fin ray 13 (Figure 13a). In culmination of this sequence, the ventral‐most IR muscle bundle originates at the base of the terminal caudal fin ray 14 and forms a connection with the neighboring procurrent ray (blue circle, Figure 13a). This connection effectively bridges the procurrent rays with the principal caudal fin ray in a similar way as on the dorsal lobe.

The configuration of IR segments in aquatic gobies is simpler, directly connecting adjacent lateral hemitrichs, with a segment crossing over at the midline to link the dorsal and ventral fin lobes (Figure 12b; Figure S4). This connecting segment always originates from the lower fin lobe, inserting onto the upper lobe's last fin ray.

In *P. argentilineatus*, the muscle bundles of the IR extend caudally to a greater extent than in their aquatic relatives, spanning nearly the full length of the unsegmented portions of the principal caudal fin rays (Figure 12d). The IR is proportionally the largest muscle of the caudal fin in *P. argentilineatus*, accounting for over one‐third (33.9%) of the total volume of the caudal intrinsic musculature (Figure S5).

The primary role of the IR bundles is to adduct the fin rays, effectively narrowing the fin web upon activation of the HL (Table 2). A study on bluegill sunfish highlighted that electrical stimulation of specific IR muscle segments in the caudal fin resulted in selective responses exclusively in the adjacent fin rays near the stimulation site (Flammang & Lauder, 2008). This indicates that individual IR segments might be controlled independently, suggesting a precise mechanism for fin movement modulation.

##### Interhemitrichialis caudalis

In *P. argentilineatus*, the lateral hemitrichs of each principal caudal fin ray are interconnected through distinct muscle bundles, leading us to designate these segments as interhemitrichialis caudalis (IH) due to their unique configuration not previously documented in the literature (Figures 12d and 13b; Table 2). Each IH segment consists of individual bundles that initiate proximally at the base of the hemitrichia and traverse the length of the fin ray to attach to the opposite hemitrich, effectively “crossing over” (Figure 13c).

We hypothesize that the IH may have evolved from the more superiorly situated IR muscle bundles, suggesting a crucial role in stabilizing the caudal fin rays during dynamic movements such as swimming, jumping, or crutching. While the IH is present in all examined aquatic gobies, these muscle bundles are less developed and exhibit a lower volume compared with those in *P. argentilineatus*, indicating a significant degree of variation in muscle development among species (Figure 13c; Figure S4; Table S2). In contrast, in the more distantly‐related zebrafish, the IH was not observed, highlighting differences in caudal fin musculature across teleosts (Figure 13c; Table S2).

### 3.4 Fin muscle properties

**FIGURE 14:**
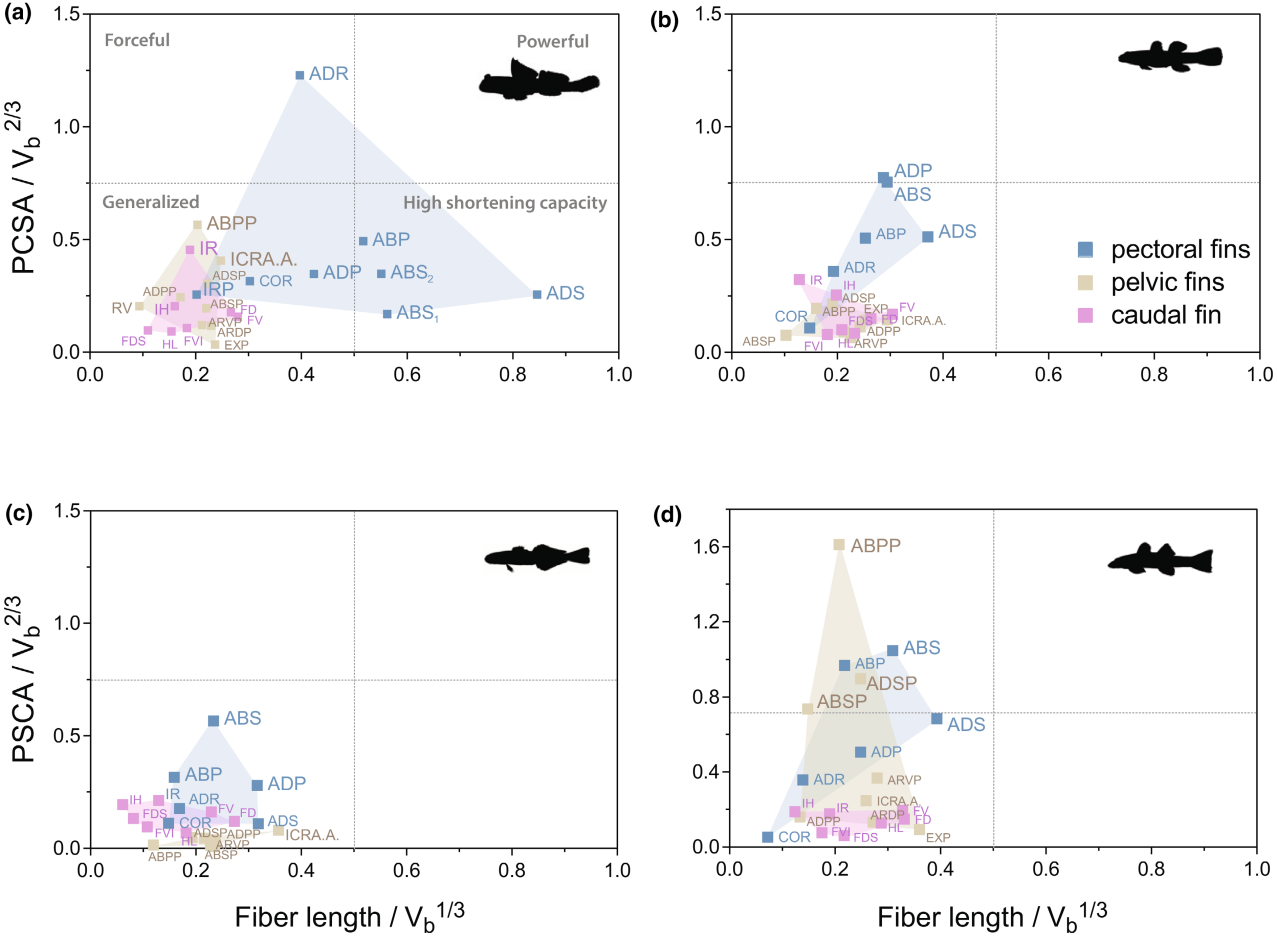
Functional space plots of locomotor fin muscles in (a) *Periophthalmus argentilineatus*, (b) *Rhinogobius brunneus*, (c) *Parioglossus dotui*, (d) *Rhyacichthys aspro*. The physiological cross‐sectional area (PCSA/*V*_b_^2/3^) and the muscle fiber length (*L*_f_/*V*_b_^1/3^) are normalized to the appropriate powers of the body volume. The horizontal dashed line suggests a gradient in fin muscles' force capability, with higher capabilities indicated above the line and lower capabilities below. Similarly, the vertical dashed line indicates a gradient in shortening capability, with higher capabilities to the right and lower to the left. The *y*‐axis for *R. aspro* is scaled differently due to the large PCSA value for the pelvic abductor muscles (ABPP). Data for deep abductor muscles in both pectoral and pelvic fins (ABP_1,2_ and ABPP_1,2_) have been combined for the analysis. For muscle abbreviations, see Table 2.

The PCSA, normalized to appropriate powers of the body volume (PCSA/*V*_b_^2/3^), for each fin muscle in *P. argentilineatus* (mean values) and individual measurements for aquatic gobies, is plotted against the fiber length, similarly normalized (*L*_f_/*V*_b_^1/3^), in Figure 14. This functional space plot provides an estimation of relative muscle function.

Muscles of the pectoral fin in *P. argentilineatus* occupy three quadrants of the functional space plot, ranging from generalized (lower left quadrant) to highly specialized muscles (upper left/lower right quadrant) (Figure 14a). For instance, the IRP exhibits a PCSA of 0.2555 ± 0.035 (mean ± SE) and the COR a PCSA of 0.3156 ± 0.029, placing them in the lower left quadrant as more generalized pectoral fin muscles in terms of muscle strength capacities (Figure 14a; Table 3). The ADR stands out as the most forceful muscle overall, with the largest force capacity (PCSA = 1.2258 ± 0.022) (Figure 14a).

**TABLE 3:** Muscle properties of paired fins and caudal fin in barred mudskipper, *Periophthalmus argentilineatus*.

Fin muscle	*V*_m_/*V*_b_	±SE	*L*_f_/*V*_b_^0.33^	±SE	PCSA/*V*_b_^0.67^	±SE
Pectoral fin						
ABS_1_	0.0953	0.021	0.5623	0.016	0.1691	0.029
ABS_2_	0.1933	0.015	0.5511	0.011	0.3483	0.019
ABP_(1,2)_	0.2573	0.023	0.5169	0.016	0.4934	0.025
ADS	0.2157	0.027	0.8457	0.040	0.2554	0.026
IRP	0.0526	0.016	0.2017	0.024	0.2555	0.035
ADP	0.1464	0.047	0.4235	0.024	0.3474	0.062
ADR	0.4883	0.008	0.3967	0.010	1.2258	0.022
COR	0.0965	0.010	0.3025	0.017	0.3156	0.029
Pelvic fin						
ABSP	0.0432	0.007	0.2201	0.012	0.1948	0.028
ABPP_(1,2)_	0.1159	0.021	0.2035	0.007	0.5650	0.088
ARDP	0.0275	0.003	0.2290	0.011	0.1175	0.019
ARVP	0.0262	0.006	0.2122	0.011	0.1208	0.022
ADSP	0.0686	0.011	0.2214	0.011	0.3057	0.033
ADPP	0.0420	0.013	0.1714	0.028	0.2438	0.062
EXP	0.0079	0.002	0.2366	0.026	0.0342	0.008
RV	0.0182	0.003	0.0935	0.017	0.2045	0.037
ICAR.A.	0.1028	0.022	0.2476	0.016	0.4062	0.061
Caudal fin						
FD	0.0469	0.001	0.2665	0.011	0.1769	0.008
FDS	0.0101	0.001	0.1096	0.012	0.0973	0.017
FV	0.0439	0.003	0.2795	0.014	0.1574	0.009
FVI	0.0192	0.001	0.1835	0.014	0.1072	0.013
HL	0.0141	0.001	0.1544	0.008	0.0920	0.005
IR	0.0853	0.012	0.1893	0.014	0.4537	0.058
IH	0.0321	0.001	0.1606	0.022	0.2045	0.020

*Note*: Muscle volume (*V*_m_), fiber length (*L*_f_) and PCSA are presented as normalized data to appropriate powers of body volume (*V*_b_), with the assumption of geometric similarity. Data for deep abductor muscles in both pectoral and pelvic fins (ABP_1,2_ and ABPP_1,2_) have been combined for analysis.

Abbreviation: SE, standard error.

The superficial and deep abductor muscles of the pectoral fin in *P. argentilineatus* demonstrate high shortening capacities with long fiber lengths and relatively high PCSAs (Figure 14a). Specifically, ABS_1_ exhibits a fiber length of 0.5623 ± 0.016 (mean ± SE), ABS_2_ has a fiber length of 0.5511 ± 0.011, and ABP_1,2_ shows a fiber length of 0.5168 ± 0.016 (Figure 14a; Table 3).

The ADS projects prominently into the lower right quadrant in Figure 14a, exhibiting long fiber lengths (ADS: *L*_f_ = 0.8457 ± 0.041), marking it as the most specialized among all the muscles examined. It demonstrates a remarkable capacity for shortening while maintaining a significantly expanded range of motion, highlighting its unique adaptability and function. The IRP exhibits the shortest fiber length among all pectoral fin muscles (*L*_f_ = 0.2017 ± 0.024). However, when compared with most of the pelvic and caudal fin muscles, the IRP presents relatively comparable fiber lengths and PCSA (Figure 14a; Table 3).

In the muscle performance space generated for the pelvic and caudal fin muscles of *P. argentilineatus*, all these muscles are located in the lower left quadrant (Figure 14a). Among the pelvic fin muscles, the deep abductor muscles (ABPP_1,2_) exhibit the highest PCSA (PCSA = 0.5650 ± 0.088), followed by the ICAR.A. (PCSA = 0.4062 ± 0.016) (Table 3), indicating a moderately strong capacity for exerting force. Specifically, the ICAR.A. facilitates pelvic fin protraction, whereas the ABPP_1,2_ contributes to fin ray adduction. This suggests that these muscles have a moderately strong capacity for exerting force, underscoring their importance in positioning the pelvic fin during terrestrial locomotion.

The normalized fiber length for pelvic fin muscles shows consistency across the spectrum, indicating uniform muscle origin and insertion points for most of these muscles (Figure 14a; Table 3). However, the mudskipper‐specific adductor RV exhibits a constrained extension range with a notably short fiber length (*L*_f_ = 0.0935 ± 0.017), despite its relatively high force capacities (PCSA = 0.2045 ± 0.037) (Table 3). Conversely, the EXP is identified as the least powerful muscle, indicating minimal force capacities (PCSA = 0.0342 ± 0.008) (Figure 14a; Table 3).

Among the caudal fin muscles, the IR (PCSA = 0.4537 ± 0.058) and IH (PCSA = 0.2045 ± 0.020), exclusive to the principal caudal fin rays, exhibit the greatest PCSAs (Table 3), highlighting their critical role in adjusting the caudal fin web during terrestrial movement.

Other intrinsic caudal fin muscles in *P. argentilineatus* show relatively modest maximum force capacities, evidenced by their lower PCSAs (Figure 14a; Table 3). The intrinsic flexor muscles attaching to the principal caudal fin rays possess the longest fibers (FD: *L*_f_ = 0.2665 ± 0.011, FV: *L*_f_ = 0.2795 ± 0.014). These two flexor muscles are responsible for lateral caudal fin movement, critical during both aquatic and terrestrial movements – activities that may include rapid escape maneuvers or the use of the tail on sloped terrains, as documented in prior studies (McInroe et al., 2016; Swanson & Gibb, 2004).

In comparison, aquatic gobies generally exhibit more generalized muscle configurations, predominantly located in the lower left quadrant of the functional space plot (Figure 14b–d). An exception is the rheophilic *R. aspro*, which is characterized by its robust ventral pelvic fin abductors (ABPP: PCSA = 1.622, ABSP: PCSA = 0.7358) and a pronounced dorsal superficial adductor (ADSP: PCSA = 0.8965) (Figure 14d; Table S1). This species also has relatively strong pectoral fin abductor muscles (ABS: PCSA = 1.0467, ABP: PCSA = 0.9684) (Figure 14d; Table S1). Despite the significant muscle capacities of *R. aspro* for both pelvic and pectoral fins, the overall low shortening capacity, due to shorter fiber lengths (Figure 14d; Table S1), contributes to reduced maneuverability when compared with the terrestrial locomotion of *P. argentilineatus*.

## 4 DISCUSSION

### 4.1 Pectoral fins

The pectoral fins of the barred mudskipper, *P. argentilineatus*, have undergone significant osteological changes to facilitate life on land. A key adaptation is the evolution of a robust shoulder girdle that provides increased areas for muscle attachment, enabling the growth of larger, more capable muscles for improved terrestrial locomotion efficiency. The radial bones of the fin are significantly elongated and connect to the shoulder girdle through a vertical hinge joint. This elongation leads to a distinct downward tilt of the entire pectoral fin, optimizing it for ground‐based locomotion. Additionally, the fin plate has developed a concave medial surface to accommodate voluminous adductor muscles, a feature that stands in contrast to the rounded and flat fin plates typically observed in aquatic gobies. Furthermore, the pectoral fin of *P. argentilineatus* is characterized by a reduced number of fin rays and asymmetry in the fin web.

During terrestrial crutching movement, the mudskipper's pectoral fins play a crucial role in weight distribution, particularly across the ventral fin rays. As the pectoral fin reaches the point of full abduction, the proximal radials protrude from the shoulder. Despite the radials limited anterior extension in reference to the shoulder joint, the fin rays can project further forward (Pace & Gibb, 2009). A substantial layer of fibrocartilage at the “elbow‐joint” embeds the fin ray bases, enabling a forward movement of the fin web and thus, longer strides. In full depression, the ventral fin rays project onto the ground over their medial ends, while the thinner fin rays at the leading edge remain pressed against the ones beneath, effectively folding the fin web. However, during swift land movements, like jumping or evading, the pectoral fins are used differently as observed by Harris (1960). For instance, prior to landing after a leap, the pectoral fins are fanned out and stretched away to stabilize the body and soften the landing impact.

In swimming fish, the bending of the fin rays is mechanically coupled to the stretching of the fin membrane. This means that the fin's curvature modulates its stiffness (Nguyen et al., 2017). Conversely, when moving on land, the mudskipper utilizes a “folded” fin web as the main support structure, all while the ventral fin rays bear the body's weight and generate forward thrust. In general, stiffness—a measure of an object's resistance to deformation under force can be altered by adding mass or changing a structure's geometry (Baumgart, 2000). For example, adding density or thickness enhances stiffness due to the material's increased ability to withstand forces. Alternatively, changing a structure's shape or curvature, like folding a flat sheet of paper into a cylinder or triangle, can significantly increase stiffness without changing its mass, demonstrating how both mass and geometry play roles in determining structural rigidity (Venkadesan et al., 2020).

The interaction between flexural stiffness, fin curvature alterations, and mass addition in the locomotor fins of mudskippers underscores the importance of their adaptation for terrestrial environments, highlighting a significant gap in our current understanding and pointing toward the need for future biomechanical studies. This investigation becomes particularly relevant when considering the unique pectoral fin morphology observed in our μCT scans of *P. argentilineatus*, suggesting that modifications in fin ray structure could play a crucial role in optimizing flexural stiffness for terrestrial locomotion.

For example, in teleost fishes, the hemitrichia of a fin ray is typically crescent‐shaped when viewed in cross‐section (Alben et al., 2007; Conway et al., 2012; Videler & Geerlink, 1986). In some benthic fishes, hemitrichia can show morphological regionalization along the proximo‐distal length of the ray. Specifically, the fin ray segments near the base are cylindrical and hollow, offering resistance to bending, while the tips remain flexible (Taft, 2011). This structural design is thought to provide advantages during substrate interaction, suggesting a functional adaptation to benthic living.

Our μCT scans of *P. argentilineatus* revealed a distinct hemitrichia shape, which to our knowledge, has not been reported in other ray‐finned fish. Closer to the fin ray base, the unsegmented hemitrichs exhibit a rounded profile with a solid, bony core. Progressing away from the base, these hemitrichs gradually revert to the crescent shape commonly found in teleosts. This discovery highlights a novel morphological variation within the fin rays of *P. argentilineatus*, suggesting an adaptation for terrestrial locomotion. Surprisingly, extinct lobe‐finned fish from the tetrapod stem group including basal rhizodonts and *Eusthenopteron foordi* shared similar fin ray morphology like the living mudskipper. The proximal region of their fin rays was unsegmented, exhibiting a rounded cross‐sectional shape (Andrews, 1985; Goodrich, 1904). Moving toward the distal ends, the rays became segmented, adopting a crescent shape form (Andrews, 1985). A recent study by Stewart et al. (2020) utilized μCT imaging to examine the pectoral fins of the living lungfish, *Neoceratodus forsteri*, and extinct lobe‐finned species with a closer phylogenetic relationship to the crown group, such as *Tiktaalik roseae*. These species also displayed similar fin ray structures, with solid and concentric configurations proximal at the fin ray base.

Comparative studies on the dermal fin ray morphology across various groups—like the sarcopterygian fish, the tetrapodomorpha, and actinopterygian fish exhibiting amphibious behaviors—will enhance our understanding of pectoral fin adaptations during the evolutionary journey from fish to tetrapod. Furthermore, such studies could reveal if the occurrence of proximal hemitrichia with solid, concentric structures in these varied lineages signifies convergent evolution, suggesting similar adaptive responses to environmental challenges despite disparate evolutionary histories.

Expanding on the specialized characteristics of mudskipper pectoral fins, we identified differences in the shape and size of hemitrich processes at their bases compared with those in aquatic gobies. Specifically, the bases of medial hemitrich fin rays in mudskippers are significantly enlarged and robust. In free‐swimming teleost fishes, the variations in the bony processes at the base of the pectoral fin are functionally related to movement. For instance, in labriform flappers, these processes are most prominent on the leading edge of the fin and taper off as they reach the trailing edge (Thorsen & Westneat, 2005). Conversely, labriform rowers exhibit larger processes on lateral hemitrichs which serve as anchor points for abductor muscles. Recent reports further highlight the presence of lateromedial asymmetry in the dermal fin ray morphology among tetrapodomorphs, and this is also observed in living stem actinopterygians like *Polypterus* (Stewart et al., 2020). This type of patterning (referred to as dorsoventral in Stewart et al. (2020)), could have played a pivotal role in the morphological evolution of paired fins. Specifically, it might have facilitated adaptations for substrate‐based loading even before the emergence of digits. Understanding this evolution becomes clearer when examining the biomechanics of fins in amphibious fish.

Regarding fin myology, the pectoral fin muscles of *P. argentilineatus* showcase significantly greater intricacy in their design compared with those of aquatic counterparts. This complexity is likely an adaptation to handle the increased demands of “anti‐gravity” support and enhanced maneuverability required for terrestrial locomotion. For example, on the lateral side of the fin, a transformation is evident where a previously single superficial muscle (Figure S2) now divides into two distinct muscles: the abductor superficialis 1 and the abductor superficialis 2 (ABS_1_, ABS_2_) (Figure 5). These superficial abductor muscles are activated in sequence from the leading edge as the pectoral fin moves forward on land, leading to an initial elevation of the fin and then to a successive flexion of the fin rays. Following this, the fin is lowered below the horizontal fin axis by the action of the deeper abductor muscles (ABP_1,2_) (Harris, 1960).

In addition to members of the *Periophthalmus* and *Periophthalmodon* genera, a similar muscle layout for the superficial abductors has been documented in *Boleophthalmus boddarti*, another terrestrial but more distantly‐related mudskipper (Eggert, 1929; Steppan et al., 2022). Therefore, the division of the abductor superficialis into two distinct muscle groups in mudskippers may represent a specialized adaptation for terrestrial locomotion. Further research is needed to determine whether this characteristic is uniform across other Oxudercidae members.

The deep abductor muscle consists of two muscle units in *P. argentilineatus*: one originating from the shoulder and the other from the proximal radials. This configuration is typical among gobies (and has also been observed in other benthic fishes like blennies (Zander, 1972)). While the two sections are still interconnected in *P. argentilineatus*, there is a noticeable trend toward differentiation: the upper part is diminishing, whereas the lower part has developed into a fusiform muscle, indicating a shift toward separation.

On the medial side of the mudskipper fin, prominent deviations from the conventional goby pectoral fin musculature are observed. These deviations include the compartmentalization of muscles, such as the ADS and the ADR, with an overall increase in pectoral fin muscle volume. Additionally, there have been considerable changes in the connective tissues, especially the fasciae, to better facilitate muscle insertions and the transmission of force to the fin rays.

In *P. argentilineatus*, the ADS has evolved into a muscle with complex functional and structural organization, deviating from its simpler form in aquatic gobies. It features the longest fibers among all the muscles examined, making it a specialized muscle with an extended working range. The muscle is specifically adapted for fine‐tuned control of the pectoral fin and for lifting the fin during terrestrial movement (Harris, 1960). The ADS is more developed in the fin's middle to ventral region, with robust muscle bundles connecting to each fin ray independently. These muscle groupings play a crucial role in reinforcing the lower fin rays when bearing the weight of the body during locomotion.

Another interesting characteristic of the ADS is the presence of tendinous material at the intra‐fin or “elbow” joint, which likely aids in the fin's bending over this joint. Eggert (1929) identified a tendon‐like ligament (“Sehnenband”) within the ADS in mudskippers, including *B. boddarti*, *P. schlosseri*, and *P. chrysospilos*. Furthermore, Eggert (1929) described specialized muscle bundles located between the fin rays across all mudskipper species he studied. Harris (1960) noted similar structures in *Periophthalmus koelreuteri* (*= barbarus*, Murdy (1989)), and these bundles were subsequently termed as interradialis pectoralis (IRP) by Winterbottom (1973). The presence of the IRP and the fascia associated with the adductor superficialis, both indicating evolutionary adaptations to terrestrial mobility, remain to be fully explored within the Oxudercidae family.

In our study, we identified the ADR as the deepest, innermost muscle of the medial pectoral fin in all examined gobies. Unlike its configuration in aquatic gobies, where it forms a relatively thin layer covering the inner surfaces of all proximal radials, the ADR *in P. argentilineatus* exhibits significant structural enhancements and volumetric expansion with concordant high PCSA (“super‐muscle”) (Figure 14). The skeletal adaptations at the intra‐fin joint underscore the muscle's efficient anchorage to the fin rays. The strategic orientation of muscle fibers and their linkage to aponeurotic fasciae enable the ADR to apply considerable pulling forces, especially affecting the middle and ventral fin rays. This muscle provides essential support against gravitational forces and contributes to the effective extension of the fin, illustrating the intricate balance between structural adaptation and functional requirements for terrestrial mobility.

To the best of our knowledge, the presence of aponeurotic fascia in living fishes has been identified uniquely in the coelacanth, *Latimeria chalumnae* (Huby et al., 2021; Miyake et al., 2016). In Latimeria, the abductor superficialis muscle's attachment to the pectoral fin's dermal rays utilizes fascia. Moreover, tendinous intersections within this muscle are recognized as a distinctive trait among living sarcopterygians (Miyake et al., 2016). The introduction of fascia likely contributed to enhanced fin rotation and overall mobility, marking a significant evolutionary step toward more versatile and efficient locomotor strategies.

Further emphasizing the evolutionary significance of musculoskeletal adaptations in mudskippers, the transition from individual tendons to a system of aponeurotic fasciae for the deeper pectoral fin adductors marks a pivotal adaptation for terrestrial activity. The “extensor complex” in *P. argentilineatus*, comprising the ADP, the ADR, and associated fascial tissues, exemplifies a remarkable degree of specialization for life on land. These anatomical changes collectively amplify the fin's effectiveness in terrestrial locomotion by ensuring a more targeted application of force along the fin's trailing edge, underscoring the complex evolutionary process from the aquatic ancestors to mudskippers.

Lastly, considering the biomechanical aspects of terrestrial mudskipper locomotion invites a comparison with the muscular activation patterns observed in aquatic fishes. Electromyography (EMG) studies have elucidated the fundamental motor pattern for pectoral fin propulsion, highlighting a sequential activation of muscle groups. Initially, the abductors are activated during the downstroke, and subsequently, the adductors are engaged during the upstroke. This sequence results in an alternating or oscillatory movement, facilitated by the antagonistic functions of the abductor and adductor muscle groups (Aiello et al., 2019; Westneat & Walker, 1997). The specialized structural arrangement of the “extensor complex” of the mudskipper fin suggests the potential for more synchronized and possibly synergistic muscle function, a hypothesis that remains to be explored through targeted EMG studies. Understanding the muscle activation in mudskippers will deepen our insight into the evolutionary innovations that have facilitated their unique niche as terrestrial locomotors among fishes.

### 4.2 Pelvic fins

Pelvic fins are used for swimming and maneuvering with complex three‐dimensional fin motions (Standen, 2008). Nevertheless, several fish lineages have exhibited variations and diversifications in their standard Bauplan, which have led to functional modifications, reductions, or even complete loss of the pelvic fin (Yamanoue et al., 2010). Within the diverse lineage of gobies, various functional changes to the pelvic fins have been observed. These fins might be entirely separated, weakly united at their bases, or completely fused, evolving into specialized suction apparatus (Maeda et al., 2017; Zander, 2011).

In our examination of mudskippers and aquatic gobies, we likewise observed significant variability in the morphological adaptations of pelvic fins, reflecting diverse alterations within the pelvic musculoskeletal system. Specifically, *P. argentilineatus* features pelvic fins with a dome‐like shape and an enlarged anterior process, accommodating large ventral abductor muscles that possess relatively high PCSA. On the other hand, the pelvic fins of rheophilic species like *R. aspro* are markedly widened and flattened, harboring exceptionally large ventral abductor muscles. The deep pelvic abductors (ABPP) were identified as the most forceful muscles among all fin muscles we studied (Figure 14). In contrast, free‐swimming gobies such as *P. dotui* display considerably reduced pelvic fins, characterized by a loss of fin rays, diminished muscle volume, and a complete absence of certain pelvic muscles. These findings generally underscore the specialized nature of pelvic fin adaptations across different goby species, and that these adaptations are finely tuned to their specific environments, whether to endure the extreme conditions found in high‐flow velocities or to meet the demands of force generation for locomotion on land.

A remarkable feature of the skeletal architecture of paired appendages in *P. argentilineatus* is the synergy between the shoulder and the pelvic girdle through a glenoid joint. This joint likely plays a pivotal role in mechanical stabilization during terrestrial locomotion, illustrating a sophisticated adaptation to land movement. A mechanical piston‐like mechanism for the pectoral‐pelvic fin antagonism has previously been suggested for *Periophthalmus gracilis* and *Periophthalmus variabilis* (Wicaksono et al., 2018). During the extension phase of the pectoral fin, responsible for propulsion on land, an inward compression of the lateral body musculature is thought to facilitate the downward movement and protraction of the pelvic fin.

Our study indicates that the ventral abductors and protractor muscles of the pelvic fins exhibit a higher force capacity compared with the dorsal adductors and retractors (Table 3). We did not include the antagonistic retractor (ICAR.M.) of the pelvic girdle in our analysis due to delineation challenges, leaving the potential retraction force of this muscle unknown. Even when considering only the abductors and adductors, the combined force capacity of the ventral abductors surpasses that of the dorsal ones, suggesting that robust pelvic fin muscles are essential in facilitating muscle‐driven abduction and the concurrent downward movement of the pelvic fin when navigating terrestrial terrains.

Among terrestrial mudskipper, the activation and coordination of the pelvic fin muscles during locomotion, as evidenced in closely related species with completely separated pelvic fins, such as *Periophthalmus barbarus* (see supplementary videos by Naylor and Kawano (2022)), synchronize with the pectoral fin during the execution of the crutching gait. Powered by the pectoral fin muscles, the propulsive moment elevates the body. Concurrently, the pelvic fins advance and raise, through the combined action of the ICAR.A., both pelvic arrector muscles, and the ventral abductor muscles. The arrector dorsalis (ARD) initiates lateral movement of the fin spine. This is followed by the arrector ventralis (ARV) guiding the spine inwards and downwards. As a result, the pelvic fin rays are shifted outward from the midline (undergoing abduction) while also advancing forward (undergoing protraction), allowing the fin web to expand fully. The fin rays, nearly equal in length, then contact the ground, aligning roughly parallel to the body axis (Figure 8d). Like the pectoral fin rays, the robust pelvic fin rays serve as mechanical supports during full abduction, bearing the body's weight as the pectoral fins begin their recovery strokes.

As the pectoral fins are lifted in preparation for the next stride, combining fin flexion and abduction, the pelvic fins may either persist in supporting the body's weight with an arched body and erected fin rays, or they might fully retract, folding beneath the body to achieve a near‐ground‐level posture. In such a retracted stance, only the head remains elevated from the substrate, as the pectoral fins progress forward during the recovery stroke. The pectoral fin rays are then positioned anterior of the shoulder at the gill level when touching the ground, which marks the initiation of the next propulsion cycle in the crutching gait.

In the terrestrial locomotion sequence of *P. barbarus*, a noteworthy observation is the preliminary retraction of the last and innermost pelvic fin rays before the pectoral fin rays contact the ground and begin to bear weight (see supplementary videos by Naylor and Kawano (2022)). The retraction, drawing the pelvic fin backward, is initiated by the activation of the EXP and RV, while the subsequent elevation of the basipterygium is primarily facilitated by the action of the ICAR.M. These muscles exert a posterior pull on the innermost fin rays, followed by the action of the ICAR.M. and the dorsal adductors. Such retraction of the pelvic fins appears to generate thrust, as the body continues to move forward even before the pectoral fins fully engage with the ground. This indicates a coordinated action between pectoral and pelvic fins, facilitating an effective transition between the lifting and thrusting phases of terrestrial locomotion and ensuring continuous forward momentum.

Although separated pelvic fins in mudskippers may enhance the range of motion and provide advantages in weight support, positioning, and climbing abilities (Hidayat et al., 2022; Wicaksono et al., 2016), this trait is not found uniformly present across all mudskipper species. It is primarily observed within specific genera of the *Periophthalmus* lineage, namely *Periophthalmus* and *Periophthalmodon* (Jaafar & Murdy, 2017).

This variability in pelvic fin configuration within mudskippers leads to questions about the role of pelvic fin musculature adaptations in their terrestrial locomotion. While one might initially hypothesize that the development of the novel RV muscle in terrestrial mudskippers is linked to the presence of unfused pelvic fins, observations from closely related species reveal a more complex evolutionary narrative. For example, *Periophthalmus modestus*, the sister species of *P. argentilineatus*, [our unpublished data], along with other terrestrial mudskippers such as *P. schlosseri*, *P. chrysospilos*, and *P. barbarus*, all display the RV muscle regardless of their pelvic fin configuration, which ranges from partially unfused to fully united (Eggert, 1929; Harris, 1960). As an adductor, the RV plays an important role in modulating the innermost fin rays during crutching locomotion as described above, and stabilizing the innermost fin rays across different terrains, irrespective of the pelvic fin's fusion state.

The diversity in pelvic musculoskeletal systems among gobies, particularly with novel traits in terrestrial mudskippers, highlights the evolutionary complexity driven by various ecological pressures. Further investigation into specific adaptations, like the presence of shoulder‐pelvic joints or the RV muscle among Oxudercidae gobies, is essential. These anatomical modifications, being distinct from ancestral features and adapted for terrestrial mobility, are indicative of evolutionary responses to terrestrial environments.

### 4.3 Caudal fin

The caudal fin represents the distal region of the vertebral axis and can be broadly categorized into heterocercal (asymmetrical) and homocercal (symmetrical) tails (Lauder, 2000). In the latter, the vertebrae do not extend into a lobe. The major evolutionary trajectory of the caudal fin structure and shape in actinopterygians has been the transition from a heterocercal to a homocercal caudal fin. Accompanying this shift was an internal reconfiguration, as well as the addition of muscular components to the caudal fin rays. These changes enhanced fin maneuverability and fostered the specialization of individual muscles for distinct kinematic roles (Flammang & Lauder, 2009).

As modern teleosts, gobies exhibit homocercal tails, and their caudal fins are typically wide and rounded. However, the caudal fin of mudskippers is often elongated with a lanceolate shape (Jaafar & Murdy, 2017; Murdy, 1989). This shape becomes even more accentuated in species belonging to the *Periophthalmus* and *Periophthalmodon* genera, in which their caudal fins are distinctively asymmetrical—a characteristic underscored by ventrally located principal caudal fin rays that are both shorter and thickened. An additional feature in *P. argentilineatus* is the presence of truncated ventral procurrent and anal fin rays (Figure 2c).

Other elements of the skeletal tail bones of *P. argentilineatus* that deviate from typical gobies include the heavy ossification of the neural and hemal spines of the penultimate caudal vertebra (pu2) and, to a lesser extent, in the antepenultimate caudal vertebra (pu3). The close approximation of the neural spines of pu3 and pu2 reinforces the strength of these vertebrae, as previously documented in members of the *Periophthalmus* genus (Ghanbarifardi et al., 2020).

Upon examining μCT images of the caudal vertebrae, we observed modifications in the neural and hemal spines of at least two caudal vertebrae preceding pu3, which are characterized by widened and truncated spines. This extensive strengthening of the posterior vertebral column might be crucial for enduring the forces exerted on the tail during vigorous jumping, as well as during landing impact, and should be investigated further.

Regarding the caudal fin's soft tissue, we discovered distinct muscles bundles situated between the lateral hemitrichia of the principal caudal fin rays, which we have named the interradialis hemitrichialis (IH) (Table 2). Micro‐CT imaging identified separated muscle segments in all gobies we studied, though these segments were considerably less pronounced in aquatic species and absent in zebrafish (Figure 13). The IH appears to be a common trait among gobies. However, to ascertain the distribution of this specialized caudal fin muscle across gobies and other ray‐finned fish will require additional computed tomography imaging studies.

Unlike the paired fins, the muscle arrangement in the caudal fin of *P. argentilineatus* closely resembles that of aquatic gobies and zebrafish, with the primary distinction lying in the volume of the musculature. While the FD typically emerges as the largest intrinsic caudal fin muscle in aquatic gobies and even zebrafish, *P. argentilineatus* presents a unique composition (Table S2). Here, the intra‐fin‐ray muscles (IR, IH) account for nearly half (46.6%) of the caudal fin's intrinsic musculature (Figure S5). This significant volume contributes to a noticeable difference in tail thickness between *P. argentilineatus* and aquatic gobies, making the IR the most potent tail muscle in *P. argentilineatus*, as indicated by its substantial PCSA (Figure 14).

On land, the considerable volume of intra‐fin‐ray muscles is instrumental in actively controlling the fin's shape, allowing for dynamic tail reshaping facilitated by the activation of the HL. Such enhanced tail musculature is vital for *P. argentilineatus* during activities like jumping or navigating inclines, providing the necessary support for these movements (Naylor & Kawano, 2022).

Specifically, during crutching movements, the critical role of a robust caudal is highlighted as the fin rays collapse, and the tail is tilted away from the ground. This action is part of the distinctive arched posture or tripodal stance in *Periophthalmus* species, which is usually maintained during strides (see supplementary videos by Naylor and Kawano (2022)). However, the tail can also be dragged along if the body momentarily flattens. The dynamic function of the caudal fin, seemingly crucial for mechanical stability, demonstrates the significant relationship between caudal fin mechanics and terrestrial locomotion efficiency. Specifically, the caudal fin's capacity for active shape adjustment and support is key to maintaining the tripodal posture, emphasizing its integral role in the adaptability of these species to terrestrial environments.

Although the reduced contact surface of a tripodal stance might offer advantages in terms of maneuverability and reduced drag, it also introduces complexities in soft substrate environments. The concentration of body weight onto three points increases pressure at these contact areas, potentially causing greater stress and wear. Additionally, the likelihood of sinking into soft substrates could compromise the animal's stability and balance, affecting its ability to navigate effectively. Given these considerations, the benefit of the *Periophthalmus* species' tripodal stance in their natural habitats remains an open question, highlighting a critical area for future research to explore the full spectrum of their locomotive adaptations and behaviors.

## 5 CONCLUSION

The transition from aquatic to terrestrial environments in modern ray‐finned fish, such as mudskippers, has driven significant evolutionary changes in their appendage musculoskeletal systems. Mudskippers demonstrate a remarkable gait synchronization on land, diverging from the axial propulsion commonly seen in other amphibious ray‐finned fish (Lutek et al., 2022). This divergence is underpinned by reinforced pectoral‐shoulder girdles, muscle diversification accompanied by volume increase, and the development of specialized aponeurotic fasciae adhering to their fin rays. These adaptations are pivotal for their effective habitation and locomotion within terrestrial environments, enabling mudskippers to generate the necessary mechanical power to overcome terrestrial gravitational forces, and are indicative of the ongoing evolutionary experimentation within the vertebrate lineage.

Advanced imaging techniques have unveiled intricacies previously beyond the visualization capabilities of early researchers like Eggert (1929) and Harris (1960). We have detailed the musculoskeletal specializations in mudskipper fins that are critical for terrestrial locomotion, showcasing the depth of evolutionary innovation required for this ecological shift.

Finally, mudskipper fins serve other functions, such as burrowing and subterranean movement within burrows, which were not explored in this study. Future research should also examine these additional functions to provide a more comprehensive understanding of mudskipper fin locomotion across different environments.

## AUTHOR CONTRIBUTIONS

MB, FZ‐K, and KM jointly conceptualized and designed the project. KM was responsible for collecting all the specimens. FZ‐K led specimen scanning, digital segmentation, analyzed data, prepared figures and tables, and drafted the initial manuscript. PP developed the animated video. All authors critically reviewed and revised subsequent drafts. The final manuscript was approved by all authors.

## FUNDING INFORMATION

This work was supported by the Nonlinear and Non‐equilibrium Physics Unit, OIST Graduate University.

## CONFLICT OF INTEREST STATEMENT

The authors declare no potential conflict of interest.

## Supporting information


Video S1.

Data S1.

## Data Availability

The CT and video data that support the findings of this study are openly available in Dryad at https://doi.org/10.5061/dryad.sqv9s4n99.
