# Supplementary material for: Anatomical insights into fish terrestrial locomotion: A study of barred mudskipper (Periophthalmus argentilineatus) fins based on μCT 3D reconstructions
Source: J Anat. 2024 Jun 6;245(4):593–624. doi: 10.1111/joa.14071 (PMC11424826; doi:10.1111/joa.14071)
Supplement: Supplementary file 2 — Data S1. [file JOA-245-593-s002.docx]

SUPPLEMENTARY MATERIAL

**Table S1** Muscle properties of paired fins and caudal fin in aquatic gobies. Fiber length (L_f_) and PCSA data are normalized to appropriate powers of body volume (V_b_), with the assumption of geometric similarity. Data for deep abductor muscles in both pectoral and pelvic fins (ABP_1,2_ and ABPP_1,2_) have been combined for analysis.


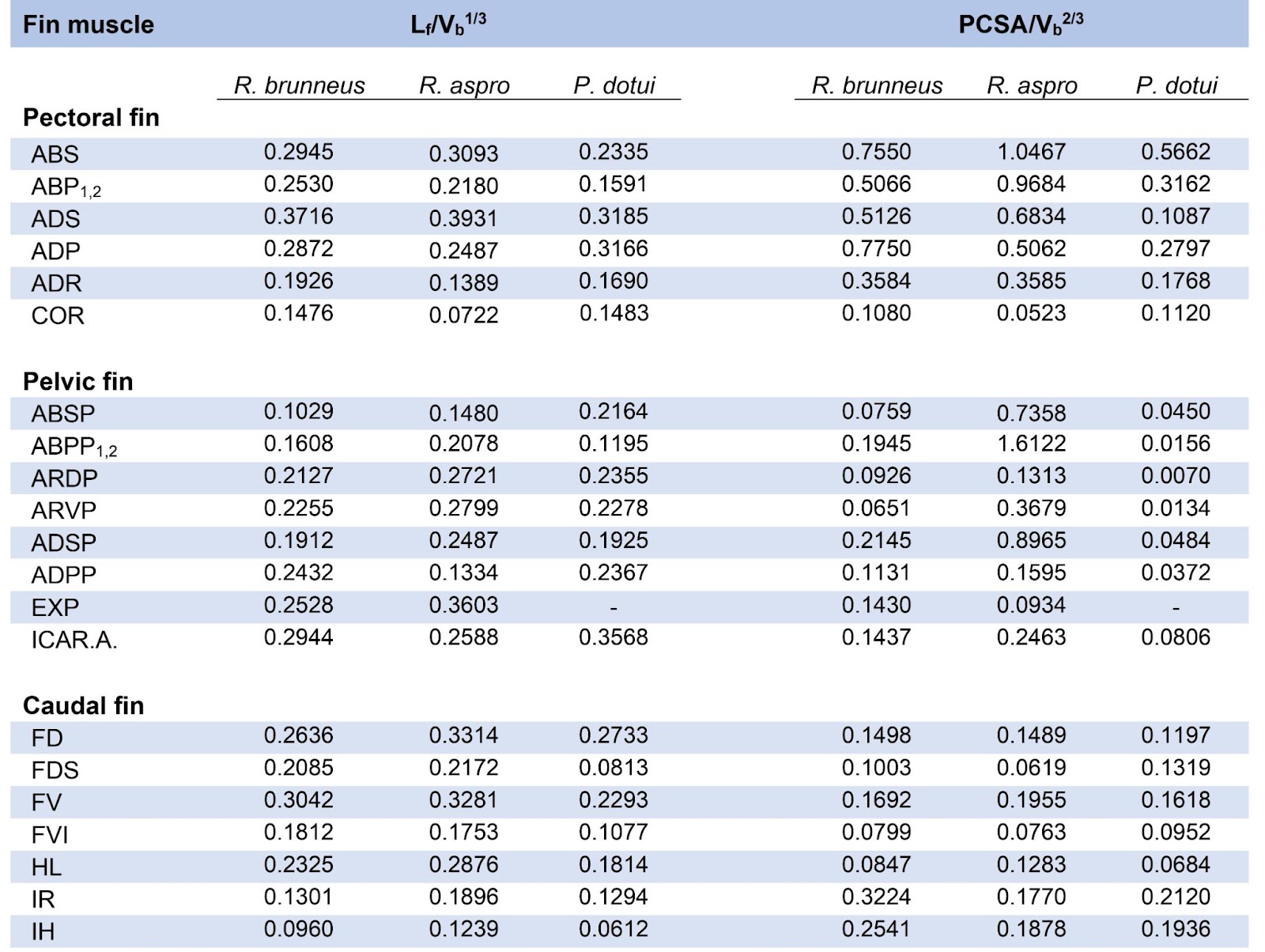


**Table S2** Summary of normalized relative muscle volumes of paired and caudal fins in all fish examined. Note: AR* refer to the arrector dorsalis and arrector ventralis muscle in the pectoral fin of zebrafish. Arrector muscles are absent in the pectoral fin of gobies. For each fish and fin, the largest muscle is indicated in bold.


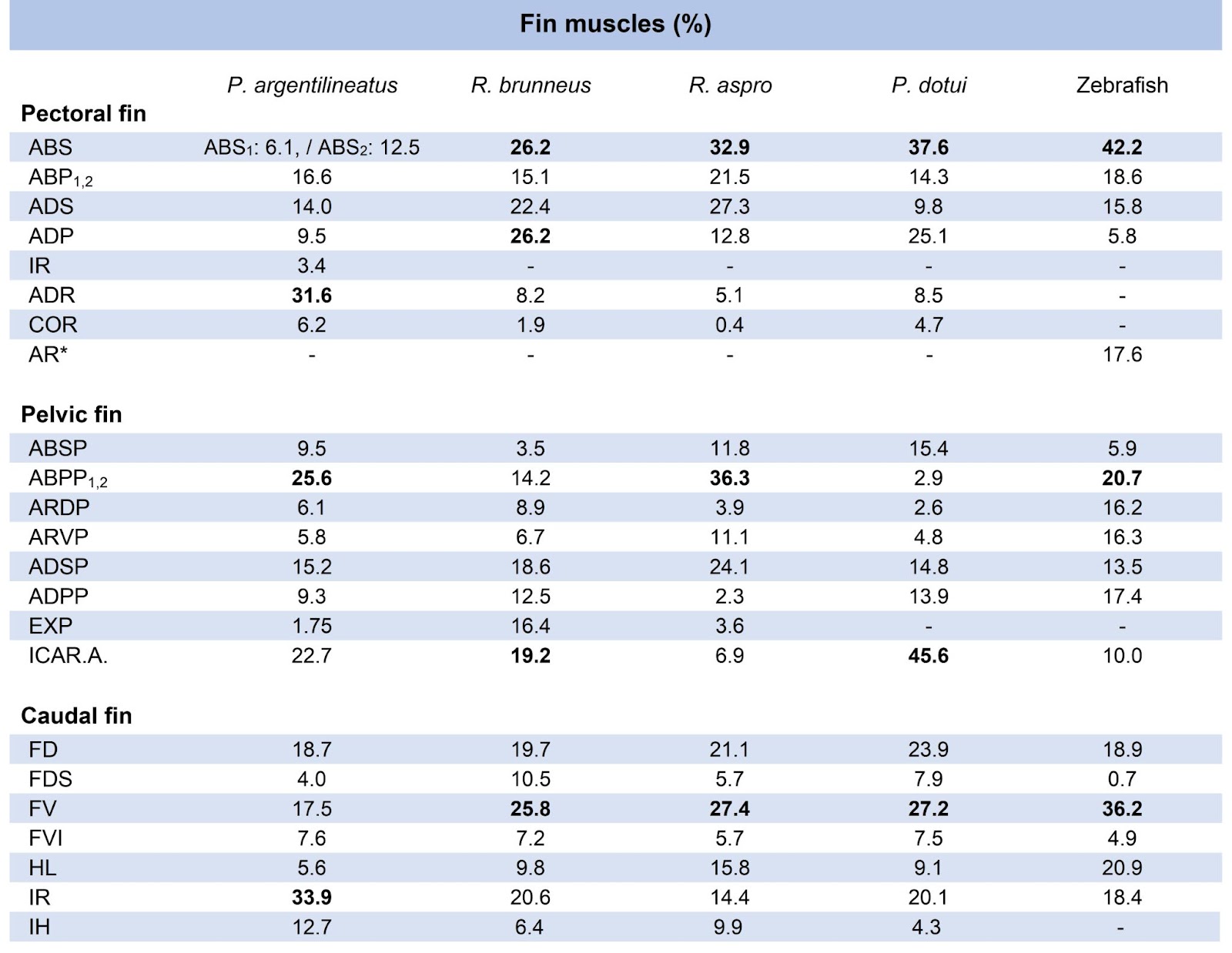


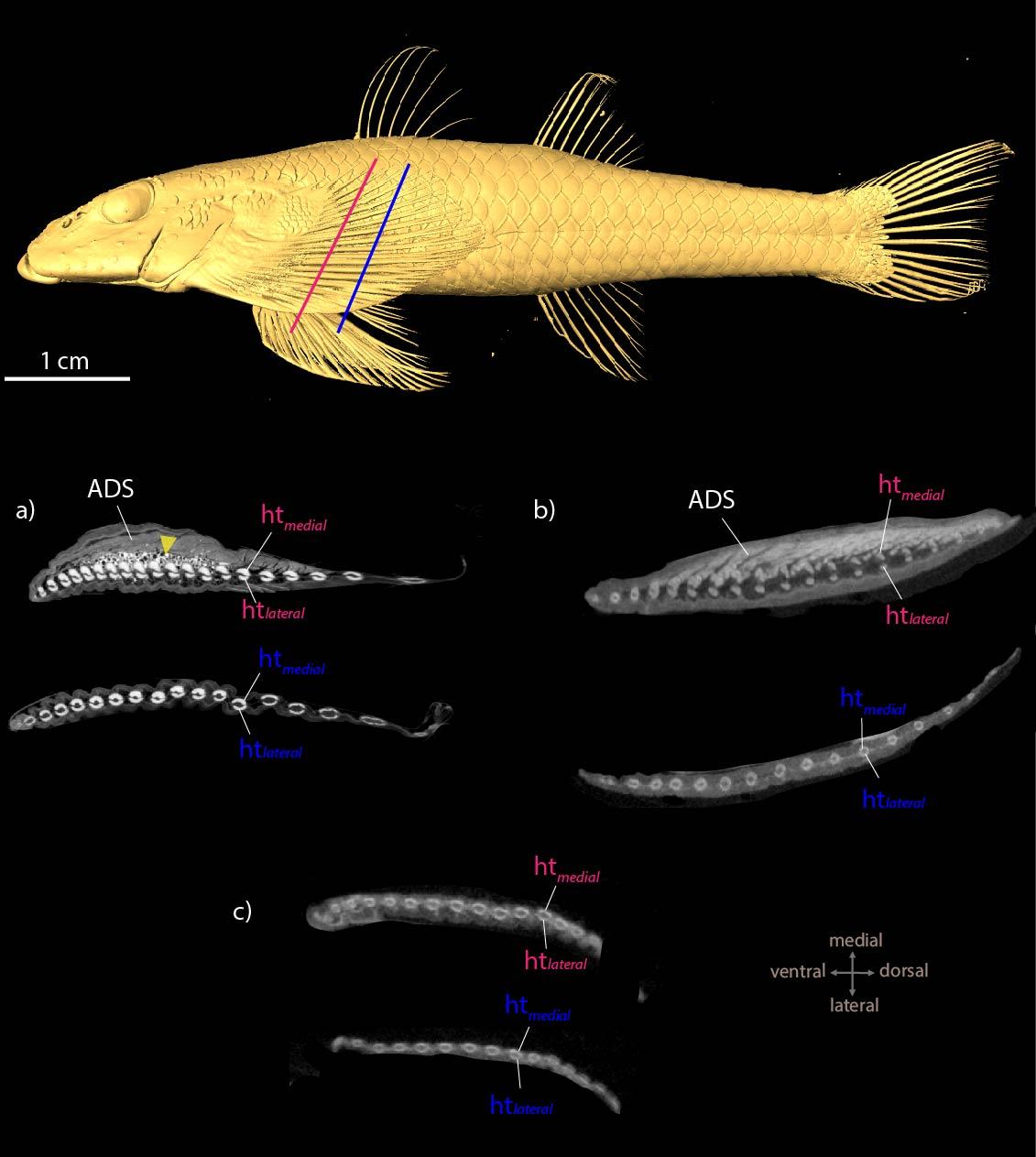


**Fig S1** Patterning of pectoral fin hemitrichia in aquatic goby representatives examined in this study. a) *Rhyacichthys aspro*, b) *Rhinogobius brunneus*, c) *Parioglossus dotui*. The isosurface represents the basal goby, *R. aspro*. In the pectoral fin of *R. aspro*, red and blue lines mark the locations of proximal and distal transections, respectively, as shown in a)-c).

**
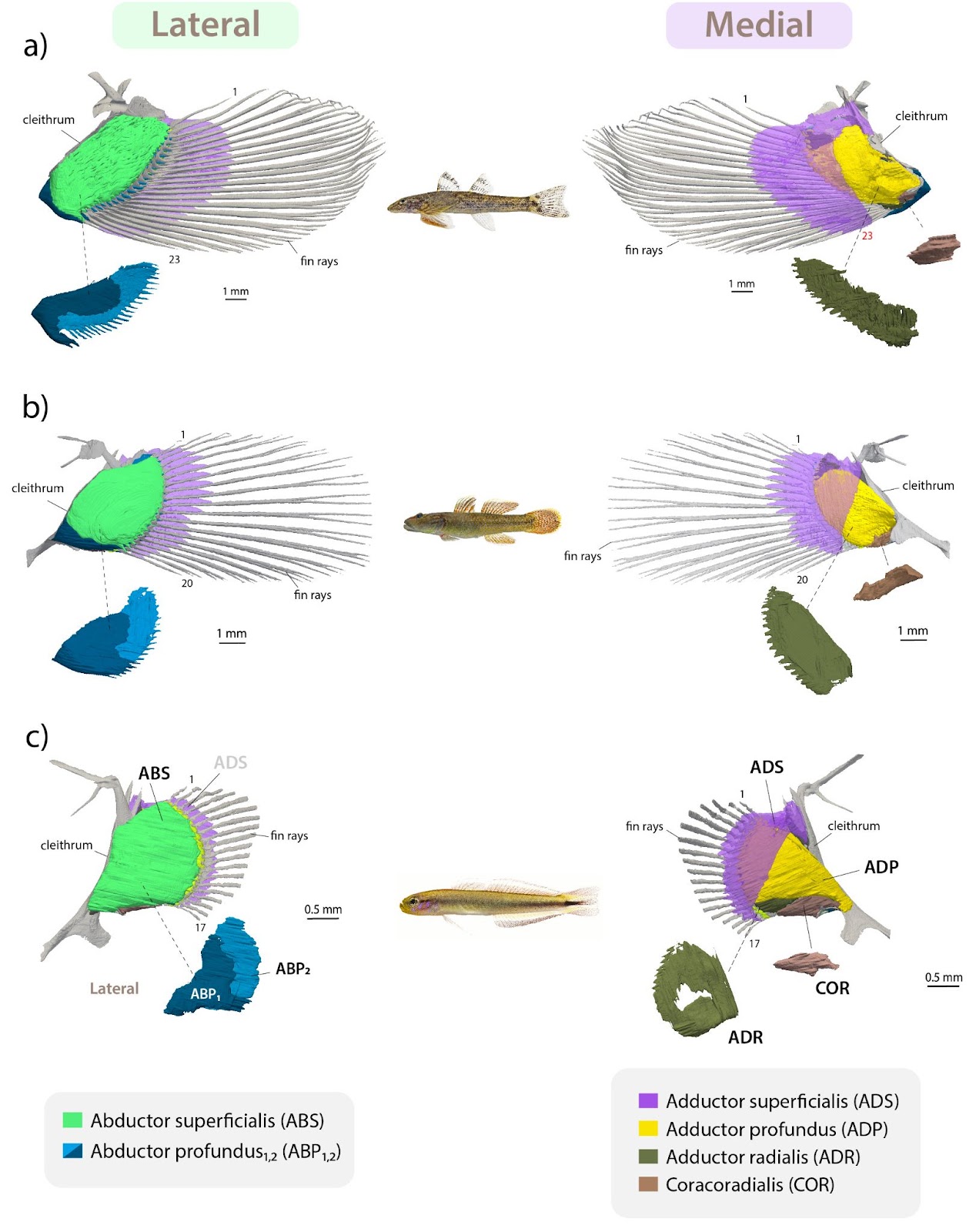
**

**Fig. S2** Pectoral fin muscles in aquatic gobies. (a) *Rhyacichthys aspro*, (b) *Rhinogobius brunneus*, and (c) *Parioglossus dotui*. Note that the individual muscles are depicted without scaling them to the entire fin for clarity. The inset provides a view of the medial pectoral fin skeleton.


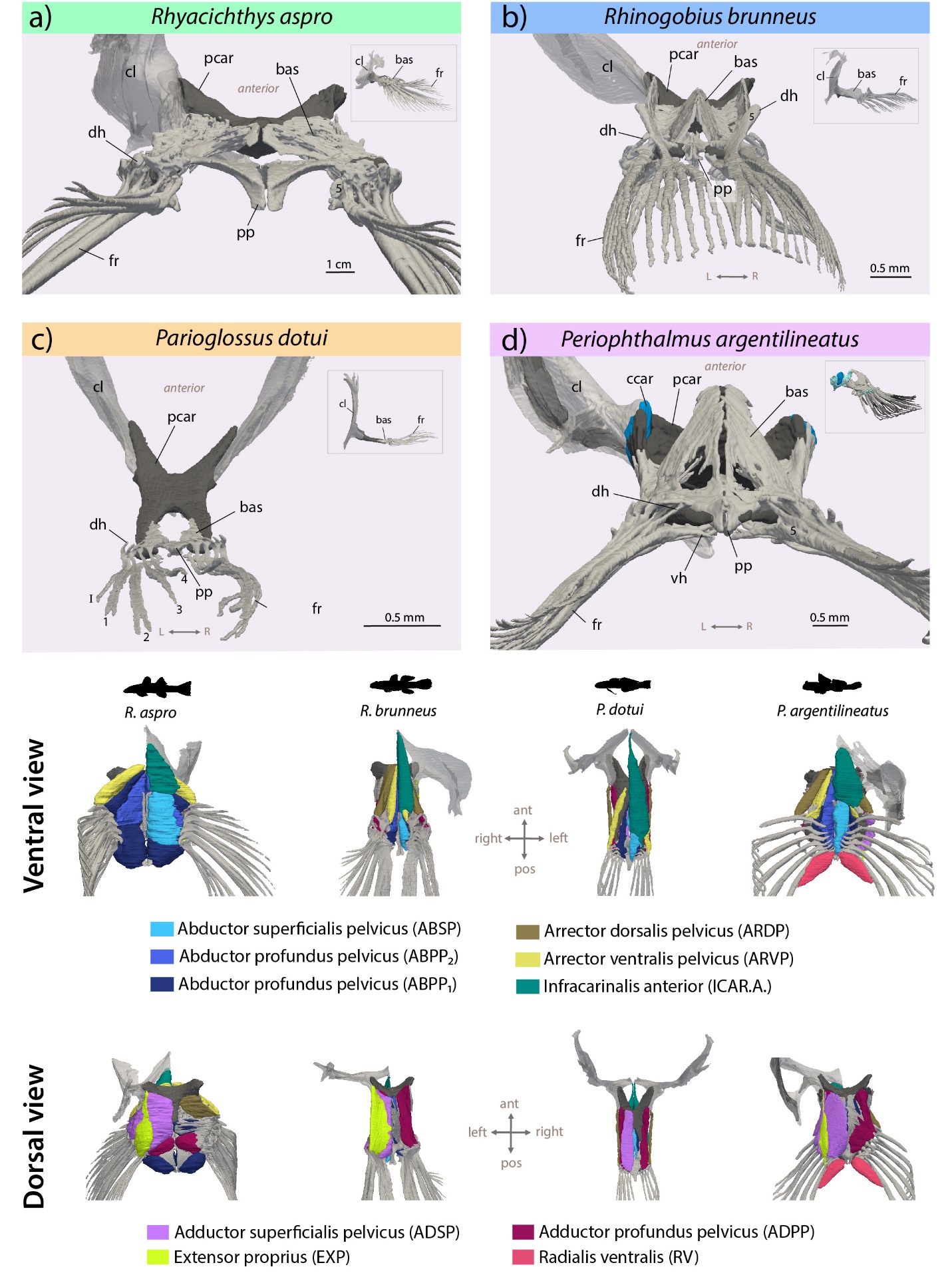


**Fig. S3** Variation in pelvic fin anatomy and associated musculature among Gobiiformes: (a-c) Aquatic gobies; (d) terrestrial mudskipper. The images in panels are viewed from the posterior, with insets in each panel providing a lateral view.

a) In *R. aspro*, the pelvic girdle is wide, robust, and heavily ossified. Each basipterygium is laterally flattened, expanding the surface area for pelvic fin muscles. Similar to *P. argentilineatus*, the pelvic fin rays are positioned consistently along the lateral aspects of the basipterygium, extending to its side, but with minimal concavity.

b) In *R. brunneus*, the pelvic bones converge to form a ridge-like pelvis. Its structure bears resemblance to that of *P. argentilineatus* but exhibits a less pronounced ventral concavity. Notably, *R. brunneus*'s pelvic fin functions as a suction disc.

c) *P. dotui* possesses a slender and flat basipterygium, characterized by a distinct 'H-shaped' pelvic cartilage. As a pelagic swimmer, this goby displays notably diminished pelvic fins.

**Ventral view**: The superficial muscle of the right basipterygium has been removed in the representations to expose the deep abductors.

**Dorsal view**: The superficial muscle of the right basipterygium has been removed in the representations to expose the deep adductor.

Abbreviations: bas - basipterygium, ccar - condylar cartilage, cl - cleithrum, dh - dorsal hemitrich, fr - fin ray, pcar - pelvic cartilage, pp - posterior process.


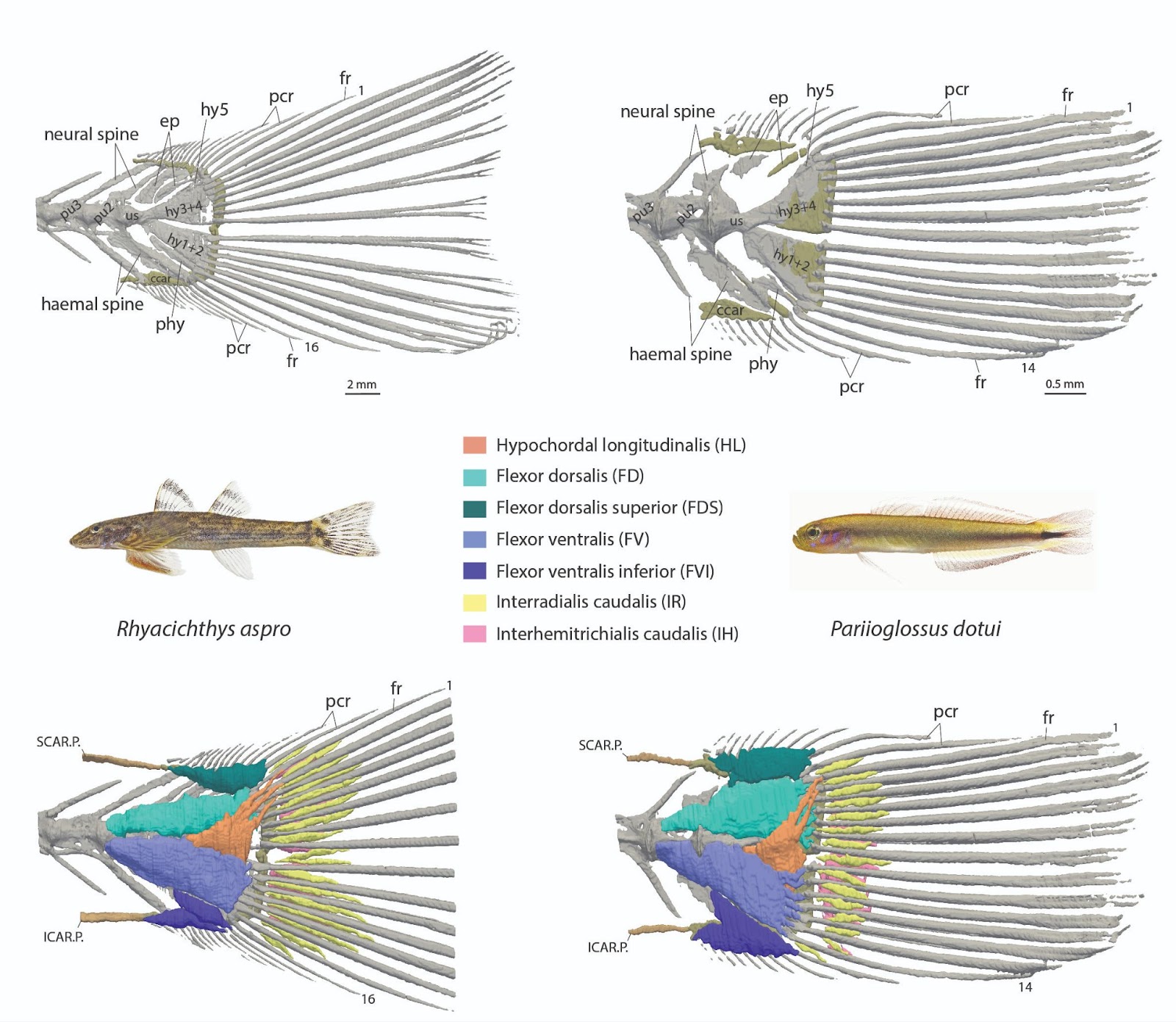


**Fig. S4** Caudal fin osteology and myology in *R. aspro* and *P. dotui*. The figure showcases the skeletal structures and muscle arrangements of the caudal fins in these two aquatic species.

Abbreviations: ccar - caudal cartilage, ep - epurals, fr - fin ray, hy - hypural, pcr - procurrent rays, phy - parhypural, pu2 - penultimate caudal vertebra, pu3 - antepenultimate caudal vertebra, us - urosyle, ICAR.P. - infracarinalis posterior, SCAR.P. - supracarinalis posterior.


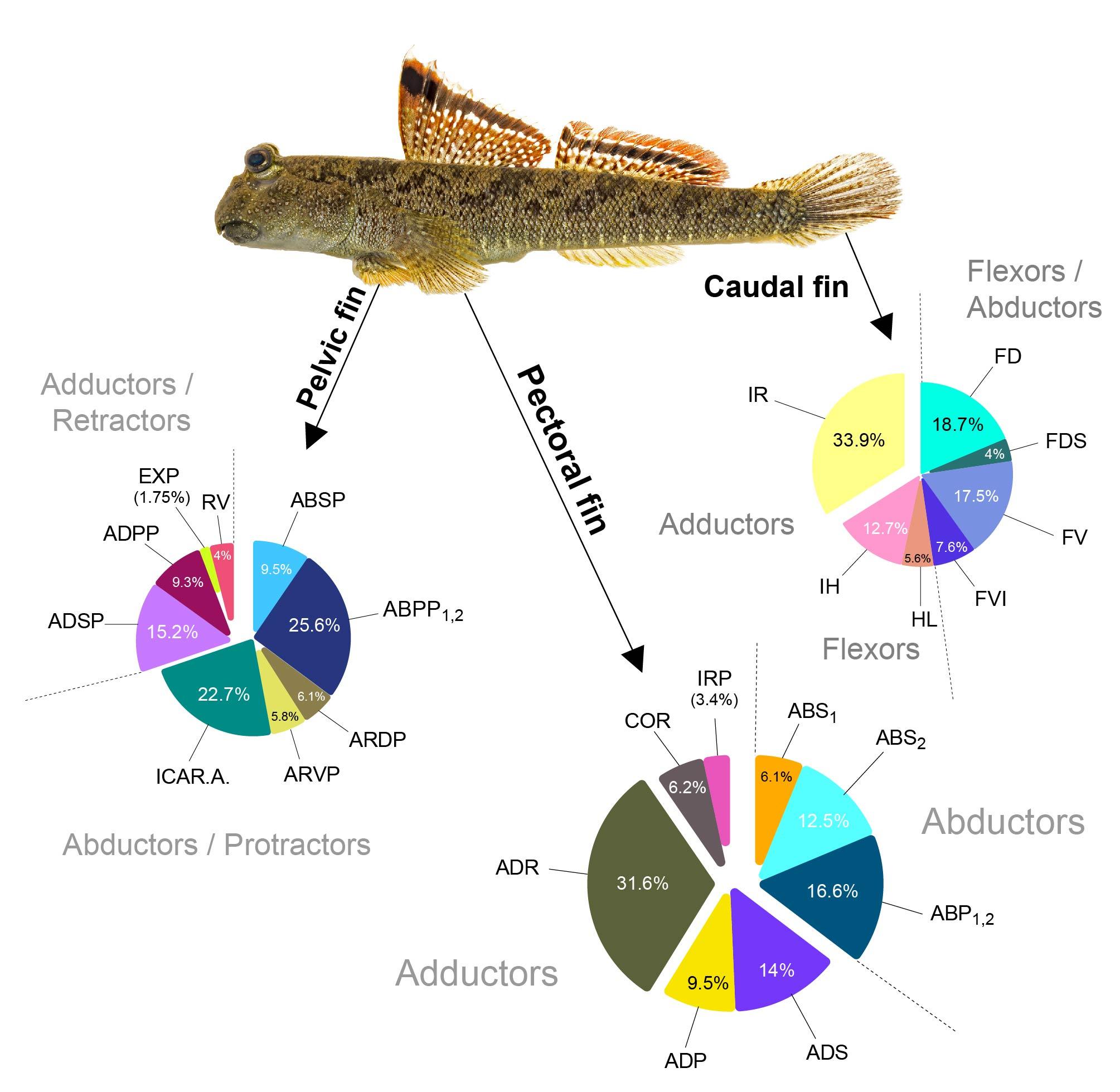


**Fig. S5** Normalized relative muscle volumes for specific muscles in the paired and caudal fins of *P. argentilineatus*. Muscles are categorized based on their primary functions during swimming and terrestrial locomotion. Abductors, which typically facilitate fin protraction (or the 'swing-phase' during crutching), and adductors, which are generally involved in fin retraction (or the 'stance-phase' during crutching), are delineated.
